# *Twist1* Suppresses Senescence Programs and Thereby Accelerates and Maintains Mutant *Kras*-Induced Lung Tumorigenesis

**DOI:** 10.1371/journal.pgen.1002650

**Published:** 2012-05-24

**Authors:** Phuoc T. Tran, Emelyn H. Shroff, Timothy F. Burns, Saravanan Thiyagarajan, Sandhya T. Das, Tahera Zabuawala, Joy Chen, Yoon-Jae Cho, Richard Luong, Pablo Tamayo, Tarek Salih, Khaled Aziz, Stacey J. Adam, Silvestre Vicent, Carsten H. Nielsen, Nadia Withofs, Alejandro Sweet-Cordero, Sanjiv S. Gambhir, Charles M. Rudin, Dean W. Felsher

**Affiliations:** 1 Department of Radiation Oncology and Molecular Radiation Sciences, Sidney Kimmel Comprehensive Cancer Center, Johns Hopkins Medicine, Baltimore, Maryland, United States of America; 2 Department of Oncology, Sidney Kimmel Comprehensive Cancer Center, Johns Hopkins Medicine, Baltimore, Maryland, United States of America; 3 Departments of Medicine and Pathology, Division of Oncology, Stanford University School of Medicine, Stanford, California, United States of America; 4 Department of Neurology, Children's Hospital Boston, Boston, Massachusetts, United States of America; 5 Department of Comparative Medicine, Stanford University School of Medicine, Stanford, California, United States of America; 6 Broad Institute, Cambridge, Massachusetts, United States of America; 7 Department of Pediatrics, Stanford University School of Medicine, Stanford, California, United States of America; 8 Department of Radiology, Stanford University School of Medicine, Stanford, California, United States of America; Cincinnati Children's Hospital Medical Center, United States of America

## Abstract

*KRAS* mutant lung cancers are generally refractory to chemotherapy as well targeted agents. To date, the identification of drugs to therapeutically inhibit K-RAS have been unsuccessful, suggesting that other approaches are required. We demonstrate in both a novel transgenic mutant *Kras* lung cancer mouse model and in human lung tumors that the inhibition of Twist1 restores a senescence program inducing the loss of a neoplastic phenotype. The *Twist1* gene encodes for a transcription factor that is essential during embryogenesis. Twist1 has been suggested to play an important role during tumor progression. However, there is no *in vivo* evidence that *Twist1* plays a role in autochthonous tumorigenesis. Through two novel transgenic mouse models, we show that *Twist1* cooperates with *Kras^G12D^* to markedly accelerate lung tumorigenesis by abrogating cellular senescence programs and promoting the progression from benign adenomas to adenocarcinomas. Moreover, the suppression of *Twist1* to physiological levels is sufficient to cause *Kras* mutant lung tumors to undergo senescence and lose their neoplastic features. Finally, we analyzed more than 500 human tumors to demonstrate that *TWIST1* is frequently overexpressed in primary human lung tumors. The suppression of *TWIST1* in human lung cancer cells also induced cellular senescence. Hence, *TWIST1* is a critical regulator of cellular senescence programs, and the suppression of TWIST1 in human tumors may be an effective example of pro-senescence therapy.

## Author Summary

Lung cancer is the most common cause of cancer death worldwide. The *Twist1* gene encodes for an essential transcription factor required for embryogenesis and overexpressed in many cancer types. It has yet to be shown *in vivo* whether *Twist1* plays a role in the initiation or maintenance of cancer. Here we demonstrate using novel transgenic mouse models that Twist1 cooperates to induce lung tumorigenesis by suppressing cellular senescence programs. Moreover, the suppression of *Twist1* in murine tumors elicited cellular senescence and the loss of a neoplastic phenotype. We found that *TWIST1* is commonly overexpressed in human lung cancers. Finally, the inhibition of *TWIST1* levels in human lung cancer cells was associated with loss of proliferation, induction of cellular senescence, and the inability to form tumors in mice. Hence, we conclude that *TWIST1* is a key regulator of cellular senescence programs during tumorigenesis. The targeted inactivation of TWIST1 may be an effective pro-senescence therapy for human lung adenocarcinomas.

## Introduction

Lung cancer is responsible for more cancer deaths in the US than colorectal, breast, and prostate cancer combined with a dismal overall survival of 15% [1]. The majority of human lung cancers are adenocarcinomas carrying somatic mutations in the genes that encode the EGFR/KRAS/BRAF pathway [2]. Observations in both experimental mouse models and human lung tumors strongly suggest that these pathways are causally responsible for lung tumorigenesis [3], [4], [5], [6], [7].

*KRAS* mutant lung adenocarcinomas are generally refractory to conventional cytotoxic therapies [8] and currently available small molecule targeted agents [9], [10]. Difficulties in pharmacologically targeting K-RAS have resulted in some labeling the protein “undruggable” [11]. Approaches such as using farnesyl transferase inhibitors to prevent prenylation of Ras for its membrane localization have not shown clinical efficacy [12], [13]. Other potential kinase targets for *KRAS* mutant tumors have been identified through RNAi screens including: TBK1, STK33 and PLK1 [14], [15], [16]. Rational candidate based approaches that target key pathways required during the process of tumorigenesis for *KRAS* mutant cancers have not been exhaustive.

One such pathway is oncogene-induced senescence (OIS), a failsafe program that prevents normal cells from progressing towards malignancy following introduction of a mutant form of an oncogene such as *Kras^G12D^* [17]. OIS is an irreversible cell cycle arrest that is characterized by cells displaying an enlarged, flattened cytoplasm, increase in senescence associated beta-galactosidase (SA-β-Gal) activity, increased chromatin condensation and changes in gene expression associated with DNA damage checkpoint proteins or cell cycle checkpoint proteins. OIS is thought to be triggered early during tumorigenesis in order to inhibit aberrant cell cycle progression, preventing pre-malignant tumors from progressing to malignancy [17]. OIS seems to be dependent on the p53-p19ARF, p16-Rb and Atf4-p27 pathways to enforce the senescent phenotype, but the requirement of any or all these pathways is highly context dependent [18], [19]. Whether these latent OIS programs can be activated in *KRAS* mutant cancers to result in a clinical effect has only recently been examined [20], [21].

Recently, Twist1, a basic helix-loop-helix transcription factor that is central to embryogenesis [22], has been shown to suppress OIS associated with *Kras^G12D^* and *EGFR2* oncogenes *in vitro* in MEFs [23] and pancreatic epithelial cells [24]. Twist1 protein expression is usually undetectable in most adult tissues, but has been shown to be overexpressed in cancers including prostate, bladder pancreatic, osteosarcomas, melanomas and breast [25], [26], [27], [28], [29], [30], [31]. The high expression of *Twist1* in cancers strongly correlates with invasive and metastatic tumor cells. *Twist1* is thought to regulate epithelial-mesenchymal transition (EMT) through the down-regulation of key proteins that maintain epithelial cell characteristics and up-regulation of proteins that confer a mesenchymal phenotype [31]. Thus, *Twist1* may act both to induce malignancies early in tumorigenesis and also promote tumor progression [32]. To date, there has yet to be reported an autochthonous model to study the role of *Twist1* overexpression in the initiation and maintenance of tumorigenesis. Here we report the generation of such a model and through this demonstration we show an important role of Twist1 in suppressing cellular senescence programs.

## Results

### Generation of an inducible lung epithelium specific *Twist1* transgenic mouse model

**Figure 1:**
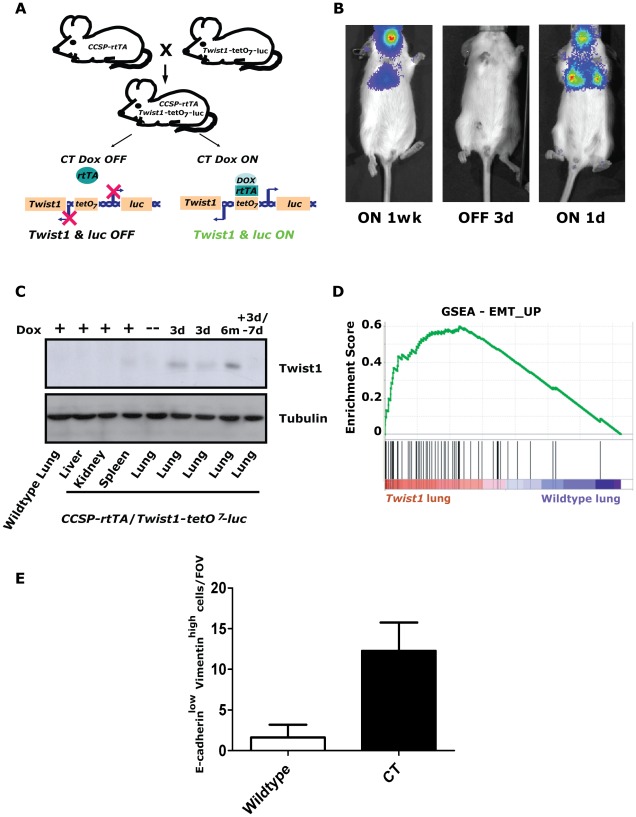
Inducible *Twist1* lung model of epithelial mesenchymal transition (EMT). (A) A mouse line containing the Clara cell secretory protein (CCSP) promoter driving the reverse tetracycline transactivating protein (rtTA) is crossed with a line containing *Twist1* and *Luc* under the control of bi-directional tetracycline-responsive elements (*tetO_7_*). In the bitransgenic animal, *CCSP-rtTA*/*Twist1-tetO_7_-luc* (CT), absence of doxycycline prevents rtTA protein from binding and activating the tetO operon. Addition of doxycycline (Dox) triggers a conformational change which enables *tetO_7_* binding, activation and *Twist1* and *luc* transcription. CT animals express Twist1 and luciferase inducibly in the lungs and trachea of bitransgenic mice as shown by (B) bioluminescence imaging (BLI) on a Xenogen Spectrum and (C) Western blotting for Twist1. BLI was performed on the same CT mouse with time “ON” or “OFF” Dox as indicated. (D) Enrichment plot of an EMT_UP signature following GSEA performed on lung mRNA samples taken from CT mouse lungs Dox ON (n = 2) and wildtype mouse lungs Dox ON (n = 2), NOM *p*-values, FDR *q*-values, and FWER *p*-values were all <0.001. (E) Plot of E-cadherin^low^-Vimentin^high^ cells per field of view immunofluorescence (IF) of the lungs from CT animals ON (n = 4) and wildtype (n = 4) animals; *p*<0.01 by Mann-Whitney t-test. d – day; wk – week; and m – month.

To produce a useful tool to address *Twist1* functions *in vitro* and *in vivo* we generated a transgenic founder line, *Twist1-tetO_7_-luc* (T), that harbored the mouse *Twist1* cDNA under the control of a bidirectional tetracycline operator sequence (*tetO_7_*) also regulating the firefly luciferase gene (*luc*) [33] (Figure 1A). This T founder was crossed to Clara cell secretory protein-reverse tetracycline transactivator protein (*CCSP-rtTA* or C) mice to generate inducible, double-transgenic (CT) mouse cohorts. CT mice contain both the rtTA activator expressed primarily in lung alveolar Type II pneumocytes [34] and the tetracycline inducible *Twist1-tetO_7_-luc* transgene allowing for spatial and temporal expression of *Twist1* and *luc* (Figure 1A).

Inducible regulation in CT mice was verified using serial small animal bioluminescence imaging (BLI) and Western blotting, respectively (Figure 1B–1C). Doxycycline drinking water given to CT mice (CT ON) induced luciferase and Twist1 expression specifically in the lung only (Figure 1C) which reverted to background luciferase and Twist1 expression by 3–7-days after doxycycline withdrawal [34], [35], (Figure 1B–1C).

To address the functional significance of ectopic *Twist1* expression in the lung epithelium global gene expression microarray analysis was performed with induced CT mouse lungs *versus* wildtype mouse lungs. Notably, after performing gene set enrichment analysis (GSEA) [36] with this dataset, we found CT ON lungs had a global gene expression pattern that had a highly significant similarity to two overlapping gene sets for EMT [37] (Figure 1D and Figure S1A) and three EMT related phenotypes (hypoxia, metastasis and wound healing [38], [39], [40]; Figure S1B–S1D). CT ON lungs showed a subset of epithelial cells appeared to lose E-cadherin and gain vimentin staining by immunofluorescence consistent with an EMT (Figure 1E and Figure S1E), strongly supportive of the gene expression data. Thus, our lung specific CT mouse model is capable of enforcing a *Twist1*-dependent transcriptional program in lung epithelial cells that is consistent with cells that have undergone EMT.

### *Twist1* accelerates *Kras^G12D^*-induced lung tumorigenesis and promotes progression to adenocarincoma

**Figure 2:**
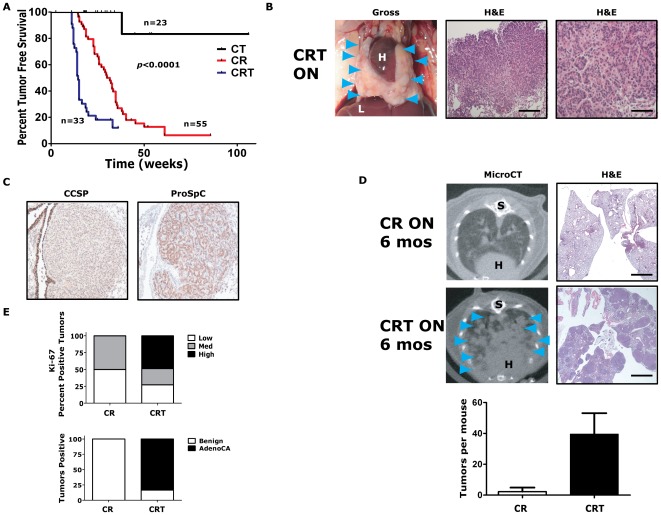
*Twist1* accelerates *Kras^G12D^*-induced lung tumorigenesis and promotes progression to adenocarcinoma. (A) Kaplan-Meier tumor free survival using serial microCT of *CCSP-rtTA*/*Twist1-tetO_7_-luc* (CT), *CCSP-rtTA*/*tetO-Kras^G12D^* (CR) and *CCSP-rtTA*/*tetO-Kras^G12D^*/*Twist1-tetO_7_-luc* (CRT) mice. The double inducible oncogene animals (CRT) developed multiple tumors at a median tumor latency that was significantly shorter than the single CR animals, 15 weeks, by log-rank analysis (*p*<0.0001). A syngenic control cohort consisting of wildtype mice, those with *tetO-Kras^G12D^*/*Twist1-tetO_7_-luc* (without *CCSP-rtTA*), *CCSP-rtTA* alone, or single oncogenes alone (n = 15 total) never developed lung tumors before 12 months of age. (B) Lung tumors from a CRT mouse at necropsy and H&E sections. Black bars equal 200 and 50 µm. H – heart; and L – liver. (C) Immunohistochemical (IHC) phenotyping of CRT tumors using antibodies against CCSP and proSpC. (D) Lung tumor burden is increased at 6 months in CRT *versus* CR mice qualitatively by microCT and H&E histology. Blue arrowheads denote lung tumors. (Lower panel) Lung tumors were quantified for CR *versus* CRT mice by microCT (n = 4 mice each). S – spine. Black bar equals 2 mm (E) Ki-67 IHC of CR *versus* CRT lung tumors (n = 3 mice each). Low - <5%; Med – 5–25%; and High - >25%. Histologic examination of lung tumors for numbers of benign lesions (hyperplasia, atypical adenomatous hyperplasia and adenomas) *versus* adenocarcinomas (AdenoCA) for CR and CRT mice (n = 2 mice each).

*Twist1* has been strongly implicated in tumor progression, but no studies have examined the effect of *Twist1* alone for autochthonous tumorigenesis. *Twist1* was not a strong oncogene when expressed alone in the lung epithelium. CT ON mice did not develop lung tumors at an increased frequency compared to wildtype FVB/N mice (Figure 2A) [41].

*Twist1* cooperated dramatically with *Kras^G12D^* expression in the lung. *CCSP-rtTA*/*tetO-Kras^G12D^* (CR) mice developed multiple synchronous lung tumors, mostly adenomas, with a median tumor latency of 32 weeks [3], [35] (Figure 2A and 2E). Triple transgenic mice, *CCSP-rtTA*/*tetO-Kras^G12D^*/*Twist1-tetO_7_-luc* (CRT), demonstrated a greatly reduced lung tumor latency compared to CR mice, 15 *versus* 32 weeks (*p*<0.0001 by log-rank analysis) (Figure 2A). CRT mice developed numerous lung tumors (Figure 2B) that appeared to be from a type II pneumocyte origin based on CCSP negative and proSpC positive immunohistochemistry (IHC) (Figure 2C). *Twist1* cooperation with *Kras^G12D^* increased the number and size of lung tumors that developed. At six months of oncogene induction there was a large difference in the total lung tumors per mouse for CRT *versus* CR, 40 tumors *versus* 2 tumors (*p* = 0.03 by *t*-test) (Figure 2D). *Twist1* co-expression with *Kras^G12D^* in the lung also appeared to promote transformation of the predominantly benign lung adenoma tumor phenotype of CR mice [3] to a malignant phenotype composed mostly of adenocarcinomas as determined by a veterinarian pathologist [42] (Figure 2E, *p*<0.0001 Fisher's exact test). A more sensitive marker of this conversion from adenoma to adenocarcinoma was the proliferative rate as we observed much higher proliferative index in CRT *versus* CR tumors (Figure 2E, *p* = 0.021 Chi-square and Figure S2A).

Although, we observed a strong genetic interaction between *Twist1* and *Kras^G12D^* for lung tumorigenesis, we did not see a pronounced effect on distant lung tumor metastases. One CRT mouse did exhibit a macroscopic metastasis to the liver confirmed by pathology (data not shown). However, in general the CRT cohort of mice (n = 33) did not demonstrate increased distant metastasis compared to CR mice (n = 55) when followed for up to 9 months of oncogene induction (data not shown). Taken together, these data suggest that *Twist1* does not appear to be a strong oncogene when over-expressed alone in the lung. *Twist1* is capable of strong cooperation with *Kras^G12D^* for lung tumorigenesis and progression. Despite markedly accelerating tumorigenesis, *Twist1* did not promote increased numbers of circulating tumors cells as detected by qPCR specific for the *luc* transgene (data not shown) and nor did *Twist1* promote distant metastasis from primary lung tumors.

### Reversible *Kras^G12D^*/*Twist1*-induced lung tumorigenesis

**Figure 3:**
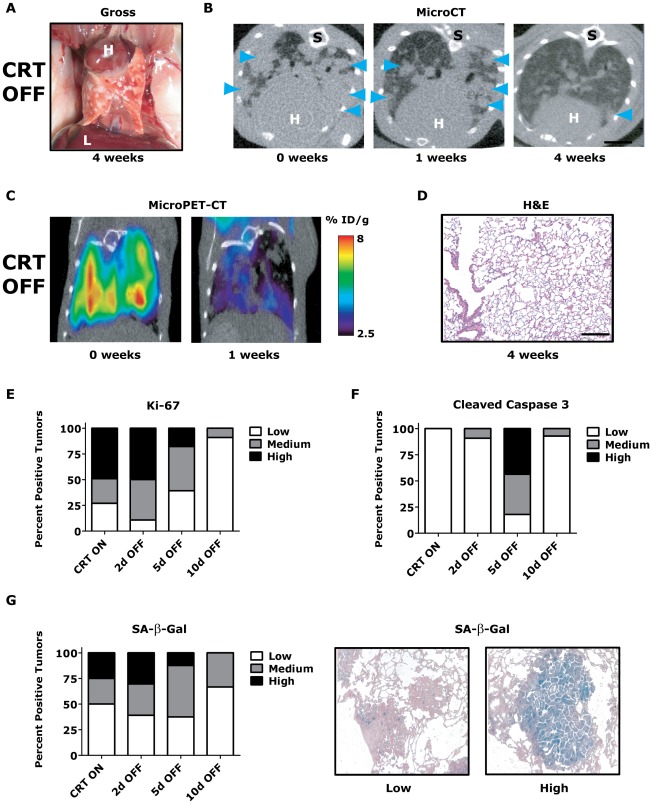
*Kras^G12D^*/*Twist1*-induced lung tumors regress following combined oncogene inactivation. (A) Gross appearance of CRT lung tumors following 4 weeks of combined *Kras^G12D^* and *Twist1* oncogene inactivation (n = 4). H – heart and L – liver. (B) Serial axial microCT of the same CRT mouse following 4 weeks of combined *Kras^G12D^* and *Twist1* oncogene inactivation (n = 4) demonstrates tumor regression. Blue arrowheads denote lung tumors. S –spine. (C) Serial coronal FDG microPET-CT demonstrate decreased metabolic tumor burden after 1 week of combined *Kras^G12D^* and *Twist1* oncogene inactivation (n = 2). (D) Normal appearing H&E histologic section from lung tumor moribund CTR OFF mouse following 4 weeks of combined *Kras^G12D^* and *Twist1* oncogene inactivation (n = 4). Black bar equals 200 µm. CRT lung tumors demonstrate (E) decreased proliferation and (F) increased apoptosis following combined *Kras^G12D^* and *Twist1* oncogene inactivation. CRT lung tumors were assayed for proliferation using Ki-67 IHC and quantified as in Figure 2E (n≥2 mice per time point). CRT lung tumors were assayed for levels of apoptosis using cleaved caspase 3 IHC and quantified (n≥2 mice per time point). Low - <1%; Med – 1–4%; and High - >4%. (G) Percentage of senescent lung tumors per mouse does not increase following combined *Kras^G12D^* and *Twist1* oncogene inactivation. The level of senescence associated-beta-galactosidase (SA-β-Gal) correlates inversely with proliferative capacity of individual tumors. CRT lung tumors were assayed for levels of SA-β-Gal and quantified (n≥2 mice per time point). Low - <10%; Med – 10–30%; and High - >30%. Representative panels of tumors with “Low” and “High” SA-β-Gal staining.

CR lung tumors were fully reversible following 2–3 weeks of *Kras^G12D^* oncogene inactivation with the mechanism of tumor regression being a combination of tumor cells undergoing proliferative arrest and apoptosis [3]. We inactivated both *Twist1* and *Kras^G12D^* from a cohort of CRT lung tumor moribund mice by the removal of doxycycline and monitored them for lung tumor regression at multiple time points using serial non-invasive imaging techniques in addition to final pathologic analysis (n = 4). CRT lung tumors showed dramatic tumor regression by gross examination (compare Figure 3A *versus* Figure 2B) that could be demonstrated serially with microCT (Figure 3B) and microPET-CT (Figure 3C and Figure S3A) after as little as 1 week of dual oncogene inactivation. By 4 weeks, CRT OFF lungs typically demonstrated no evidence of viable tumor cells on histologic analysis (Figure 3D) even despite CRT mice having considerably more advanced lung tumors than CR at similar time points (Figure 2D). CRT mice with heavy initial tumor burden did have residual fibrotic scars remaining (white spots in Figure 3A and trichome collagen staining in Figure S3B).

To gain insight into the mechanism of tumor regression, we performed a time course analysis of CRT OFF lung tumors during the first week of oncogene inactivation. CRT OFF lung tumors demonstrated a prominent decrease in proliferation and increase in apoptosis following 5 days of doxycycline withdrawal as measured by Ki-67 and cleaved caspase 3 (CC3) IHC, respectively (Figure 3E–3F, *p*<0.0001 Chi-square for both Ki-67 and CC3 and Figure S3C–S3D). As mentioned previously, *Twist1* has been shown *in vitro* to suppress *Kras^G12D^* oncogene-induced senescence (OIS) [23]. However, we did not see any appreciable increase in senescence associated beta-galactosidase (SA-β-Gal) staining following simultaneous inactivation of *Twist1* and *Kras^G12D^* in CRT OFF lung tumors (Figure 3G, *p* = 0.68 Chi-square) or by assessing for markers of cell cycle arrest (data not shown). These data suggest that although CRT lung tumors demonstrate more aggressive histologic appearance than CR tumors, CRT lung tumors are still strictly dependent on initiating oncogenes for tumor maintenance. Furthermore, *Twist1* did not alter the mechanism of tumor regression between CR OFF and CRT OFF lung tumors.

### Induction of cellular senescence in *Kras^G12D^*-induced lung tumors by inactivation of *Twist1*

The strong dependency or addiction of *Kras^G12D^*-initiated lung tumors for *Kras^G12D^* [3], [35] may have precluded us from observing any activation of OIS in CRT OFF lung tumors. Furthermore, given the genetic configuration of the CRT mouse model we were not able to examine the effects on lung tumors following inactivation of *Twist1* alone.

We addressed *in vitro* whether activation of *ras^G12V^*-induced senescence could be driven by inactivation of *Twist1* by using mouse embryonic fibroblasts (MEFs) generated from *β-actin-rtTA/Twist1-tetO_7_-luc* (BT) mice. BT MEFs were shown to be inducible with doxycycline *in vitro* by Western blotting (Figure S4A). As reported previously [23], we found *Twist1* was able to fully suppress *ras^G12V^*-induced senescence *in vitro* as shown by proliferation curves and SA-β-Gal staining (Figure S4B–S4D). We removed doxycycline from the media of BT MEFs infected with *ras^G12V^* to downregulate expression of *Twist1* at Day 12. These de-induced BT MEFs activated OIS *in vitro* as shown by decreased proliferation and increased SA-β-Gal staining relative to cells maintained in the presence of doxcycline (*p* = 0.0025 for proliferation and *p* = 0.0294 for SA-β-Gal; Figure S4E–S4F). These data suggested that at least *in vitro* inhibition of *Twist1* can activate *ras^G12V^*-induced senescence.

**Figure 4:**
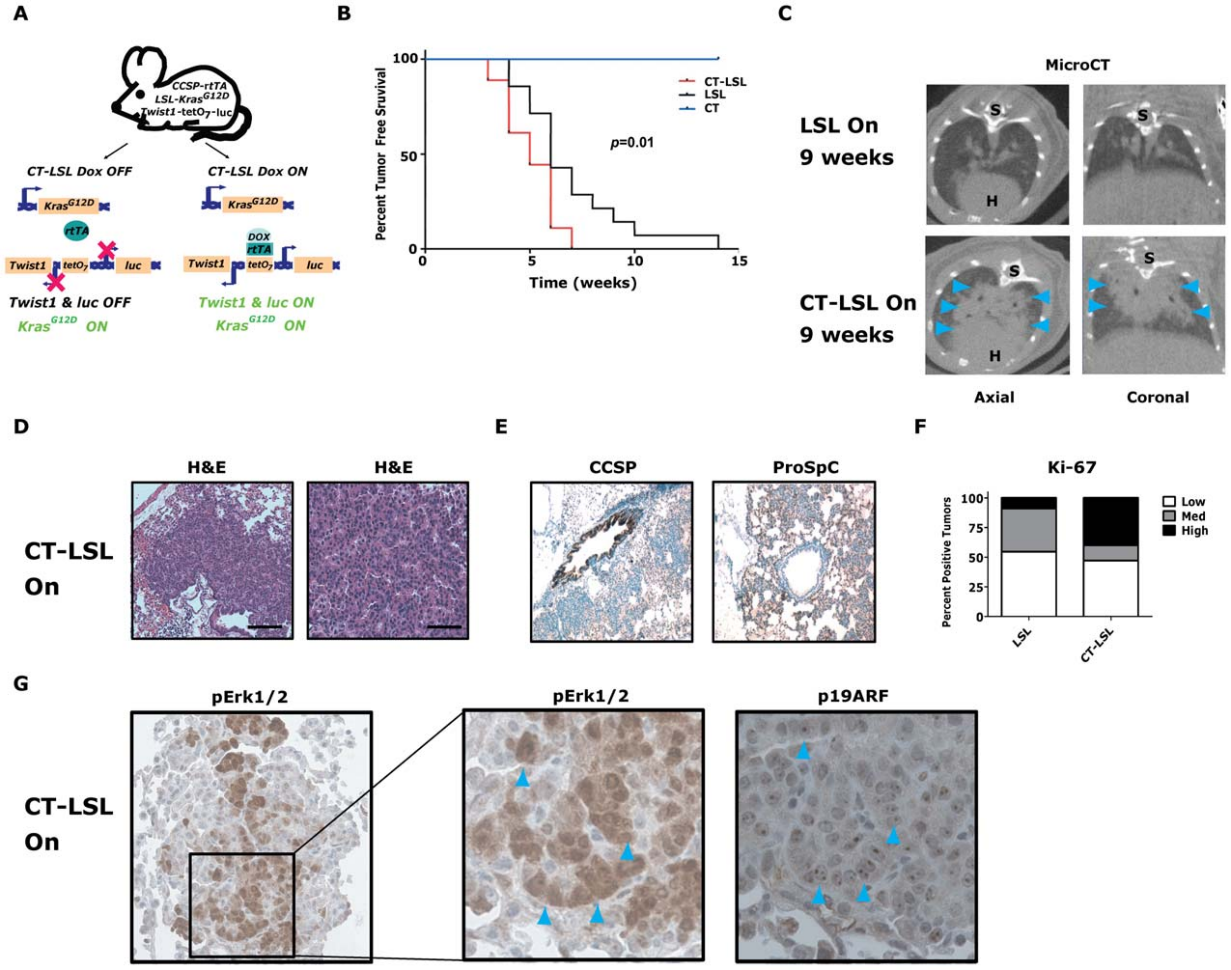
*Twist1* accelerates conditional *Kras^G12D^*-induced lung tumorigenesis. (A) Crosses (CT×LSL) to produce *CCSP-rtTA*/*Twist1-tetO_7_-luc*/*LSL-Kras^G12D^* (CT-LSL) mice. CT-LSL mice are infected with intranasal Cre to activate *Kras^G12D^*. Addition of Dox enables *Twist1* and *luc* transcription. In contrast to CRT OFF mice, CT-LSL OFF mice have *Kras^G12D^* still active and only *Twist1* expression is inactivated. (B) Kaplan-Meier tumor free survival by serial microCT of F1 littermates with CT, LSL and CT-LSL genotypes. The double oncogene animals (CT-LSL, n = 18) developed multiple tumors at a median tumor latency that was significantly shorter than the single LSL (n = 14) animals, 5 *versus* 6 weeks (CT-LSL *versus* LSL, by log-rank analysis *p* = 0.0121). CT animals (n = 17) and littermate controls not infected with AdCMVCre (n = 5) never developed lung tumors. (C) Lung tumor burden is increased at 9 weeks post-AdCMVCre in CT-LSL *versus* LSL mice qualitatively by representative microCT. Blue arrowheads denote lung tumors. H – heart; and S – spine. (D) H&E stained sections of lung tumors from a CT-LSL mouse. Black bars equal 200 and 50 µm. (E) Immunohistochemical (IHC) phenotyping of CT-LSL lung tumors indicate a type II pneumocyte cell of origin using CCSP and proSp-C markers. (F) Ki-67 IHC of LSL *versus* CT-LSL lung tumors (n = 2). Low - <5%; Med – 5–25%; and High - >25%. (G) pErk1/2 and p19^ARF^ IHC staining in serial sections demonstrate overlap. Note the nucleolar staining of p19^ARF^, specific nuclei are denoted by blue arrowheads.

To examine whether *Twist1* inhibition could be a viable therapeutic target *in vivo* for *Kras* mutant autochthonous lung cancers, we generated mice in which only *Twist1* expression was doxycycline-dependent (Figure 4A). The *LSL-Kras^G12D^* (LSL) model allows for conditional activation of an endogenous *Kras^G12D^* allele in the lungs following intranasal adenoviral delivery of Cre recombinase (AdCMVCre) [43]. The strain background difference between CT (FVB/N) and LSL (C57BL/6) transgenic models forced us to use first generation progeny from these crosses for all our experiments. We generated tri-transgenic CT-LSL animals (Figure 4A), activated *Twist1* expression with doxycycline, then conditionally activated the *Kras^G12D^* allele with AdCMVCre and followed these CT-LSL ON mice and similarly treated littermate controls for lung tumor development. *Twist1* accelerated conditional *Kras^G12D^*-induced lung tumorigenesis in CT-LSL mice (CT-LSL *versus* LSL, *p* = 0.0121 by log-rank analysis, Figure 4B–4C, similar to CRT mice, although to a lesser degree. CT-LSL lung tumors were similar to CRT tumors based on histology, expression of type II pneumocyte markers, and increase in the proportion of lung tumors with a higher proliferative index (Figure 4D–4F, *p*<0.0001 Chi-square). Recently, two groups have demonstrated in a similar LSL-p53 model system that p19^ARF^ is a critical sensor of oncogenic stress from MAPK signaling in adenocarcinomas [44], [45]. We similarly observed overlap of activated p19^ARF^ (nucleolar localization) with areas of intense pErk1/2 staining by IHC in our CT-LSL ON tumor model (Figure 4G).

**Figure 5:**
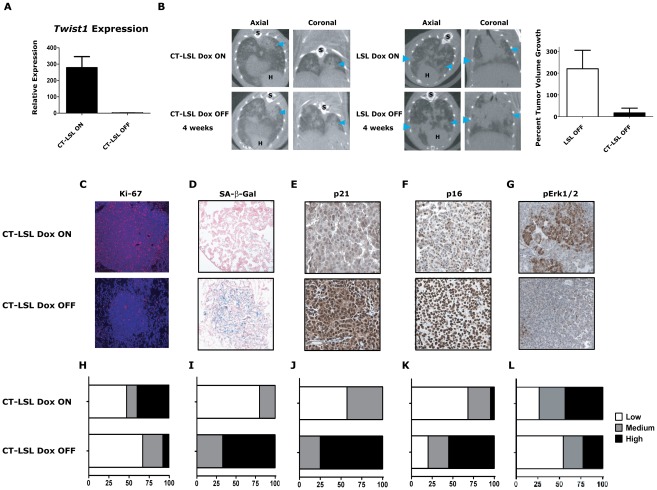
Activation of *Kras*-induced senescence by down-regulation of *Twist1* in autochthonous *Kras^G12D^*/*Twist1*-induced lung tumors. (A) Verification by qPCR that *Twist1* mRNA levels are reduced following doxycycline withdrawal in CT-LSL OFF (n = 4) compared to CT-LSL ON (n = 3) lung tumors. (B) CT-LSL OFF lung tumors are static following single *Twist1* inactivation. Representative serial microCT of CT-LSL lung tumor moribund mouse just before, CT-LSL ON, and 4 weeks following doxycycline removal from the drinking water, CT-LSL OFF, resulting in de-induction of *Twist1*only (n = 13 tumors quantified from 4 mice). For comparison, *LSL-Kras^G12D^* (LSL) mouse lung tumors grow despite withdrawal of doxycycline, LSL OFF (n = 11 tumors quantified from 3 mice). Percent tumor volume growth was quantified and calculated showing CT-LSL OFF tumor stasis after 4 weeks compared to LSL OFF (*p*<0.0001). H – heart; and S – spine. CT-LSL OFF lung tumors demonstrate markers consistent with an increase in the number of senescent cells, such as (C) reduction in proliferation by Ki-67 IHC, (D) increased lung tumors positive for SA-β-gal staining, increased levels of (E) p21 and (F) p16 by IHC (n = 3 mice per staining). (G) pErk1/2 levels reduced moderately following *Twist1* inactivation in CT-LSL OFF tumors. (H–L) Quantification of (C–G) as described in previous figures for Ki67 (see Figure 2) and SA-β-gal (see Figure 3) staining; and 21, p16 and pERk1/2 as follows - Low - <10%; Med – 10–25%; and High - >25%. All animals in these experiments were taken off doxycycline (“OFF”) continuously for 2–5 weeks and then sacrificed.

We next inactivated the expression of *Twist1* alone in a cohort of CT-LSL lung tumor moribund mice by withdrawal of doxcycline (CT-LSL OFF, n = 4). *Twist1* levels were confirmed in CT-LSL OFF tumors by qPCR (Figure 5A, *p* = 0.004 by *t*-test) and by serial BLI (data not shown) to return to levels in wildtype lungs. Interestingly, CT-LSL OFF lung tumors showed tumor stasis by serial microCT over the course of the 4 weeks of *Twist1* inactivation in stark contrast to the progressive tumor growth seen for the control LSL OFF tumors (Figure 5B; 18% *versus* 220% growth, *p*<0.0001 *t*-test).

To further characterize in an unbiased manner the mechanism by which *Twist1* suppression was inducing tumor stasis we performed microarray analysis. We compared CT-LSL OFF lung tumors with normal lung and microdissected lung tumors from CR, CRT, LSL, CT-LSL ON and CT-LSL OFF mice. The analysis of 2,163 annotated pathways using single sample GSEA (ssGSEA), an algorithm designed for modest samples sizes [as used previously in [16]], found gene sets representing p21 ectopic overexpression to be highly correlated with the CT-LSL OFF lung tumor transcriptional program (Figure S5A–S5B). We used the complimentary Ingenuity Pathway Analysis (IPA) to identify canonical pathways from the differentially expressed genes between CT-LSL ON and CT-LSL OFF tumors. Consistent with ssGSEA we found Twist1 regulated key drivers of cellular senescence (genes encoding p21, p16, p27 and IL-6) and EMT (genes encoding cadherins, vimentin and alpha-catenin) in the context of *Kras^G12D^*-driven lung tumors (Figure S6). Directed IHC analysis of CT-LSL OFF tumors confirmed ssGSEA and IPA that molecular changes consistent with activation of OIS, such as marked decreases in proliferation by Ki-67 and pronounced increases in staining for SA-β-Gal, p21 and p16 (Figure 5C–5F and 5H–5K, *p*<0.0007 Chi-square for 5H–5K).

The single inactivation of *Twist1* in our CT-LSL OFF tumors also appeared to decrease the number of adenocarcinomas as shown with decrease in tumors with high proliferative rate to a frequency similar to LSL alone (compare Figure 5C and 5H to LSL from Figure 4F, *p* = 0.93 Chi-square). In addition MAPK signaling intensity decreased in CT-LSL OFF significantly compaed to CT-LSL ON (Figure 5G and 5L, *p*<0.015 Chi-square). The decrease of highly proliferative adenoncarcinomas with active MAPK signaling in *Kras^G12D^*-induced lung tumors was also seen following p53 restoration [44], [45]. Lastly, apoptosis increased only very slightly in a subset of the CT-LSL OFF tumors as demonstrated by cleaved caspase 3 IHC (data not shown). These data provide the first *in vivo* evidence that *Kras* mutant lung adenocarcinomas can be clinically impacted by activating a latent program of cellular senescence *via* the inhibition of *Twist1*.

### *TWIST1* is commonly overexpressed in human lung cancers

**Figure 6:**
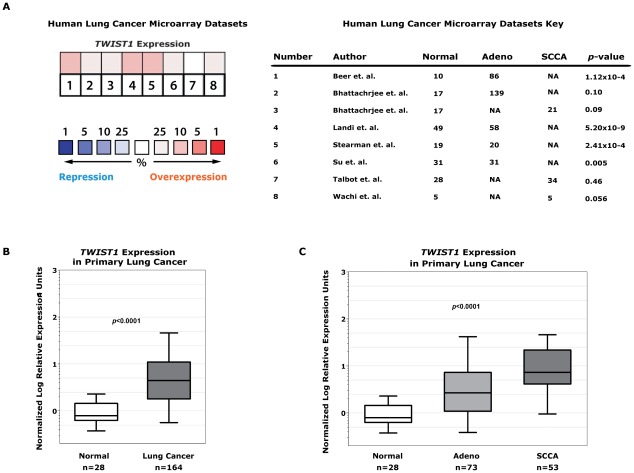
*TWIST1* is overexpressed in human primary lung cancers. (A) Human non-small cell lung cancer samples (n = 394) compared against normal lung (n = 159) from seven independent microarray datasets for *TWIST1* expression using Oncomine. The heatmap contains individual studies (see accompanying legend; #2 and #3 are from the same dataset analyzed by adenocarcinoma and squamous cell carcinoma, respectively). The heat map intensity corresponds to percentile overexpression (red) or repression (blue). The median rank across all eight datasets demonstrates *TWIST1* is overexpressed in human lung cancer, *p* = 0.04. (B) We validated this microarray analysis by performing qPCR on primary human tumor samples for *TWIST1*. *TWIST1* mRNA is overexpressed in human lung cancer (n = 164) compared to normal lung (n = 28), *p*<0.0001 by Mann-Whitney t-test. (C) Analysis of data from (B) broken down by adenocarcinoma (Adeno, n = 73) and squamous cell carcinoma (SCCA, n = 53) histology, *p*<0.0001 using one-way ANOVA.

The relevance of *TWIST1* as a potential therapeutic target in human lung cancers was evaluated by examining public gene expression microarray datasets. We found seven independent human lung cancer gene expression datasets that in total consisted of 394 tumor samples and 159 normal lung samples [46], [47], [48], [49], [50], [51], [52] (Figure 6A). Six out of the 7 datasets, as well as aggregate analysis of all 7 datasets demonstrated *TWIST1* overexpression in lung cancers (*p* = 0.04 for aggregate). The analysis included tumors of adenocarcinoma and squamous cell carcinoma histology which comprise the two most common subtypes encountered in human lung cancer.

This microarray expression data was directly validated using quantitative PCR (qPCR) for *TWIST1* on human lung cancer samples. In total we screened by qPCR 164 human lung tumor samples and confirmed that *TWIST1* was indeed overexpressed (100/164 or 61% demonstrate at least 3-fold upregulation, 43/164 or 26% at least 10-fold overexpression and in some cases as high as 536 fold overexpression was observed, *p*<0.0001 by *t*-test; Figure 6B). *TWIST1* was similarly overexpressed in all the histologies examined including adenocarcinoma and squamous cell carcinoma (*p*<0.0001 by ANOVA) (Figure 6C). The range of relative *TWIST1* overexpression observed by qPCR in our 164 primary human lung cancer samples (range 3–536 fold *TWIST1* overexpression) was similar to the *Twist1* overexpression observed in our mouse *Kras^G12D^*-*Twist1*-induced lung tumors (range 5–960 fold *Twist1* overexpression, n = 6). Together these data demonstrate that *TWIST1* is commonly overexpressed in human lung cancer and that our *Kras^G12D^*-*Twist1* mouse models do reflect human lung cancer.

### Activation of cellular senescence in human *KRAS* mutant lung cancer cells by targeting *TWIST1*

**Figure 7:**
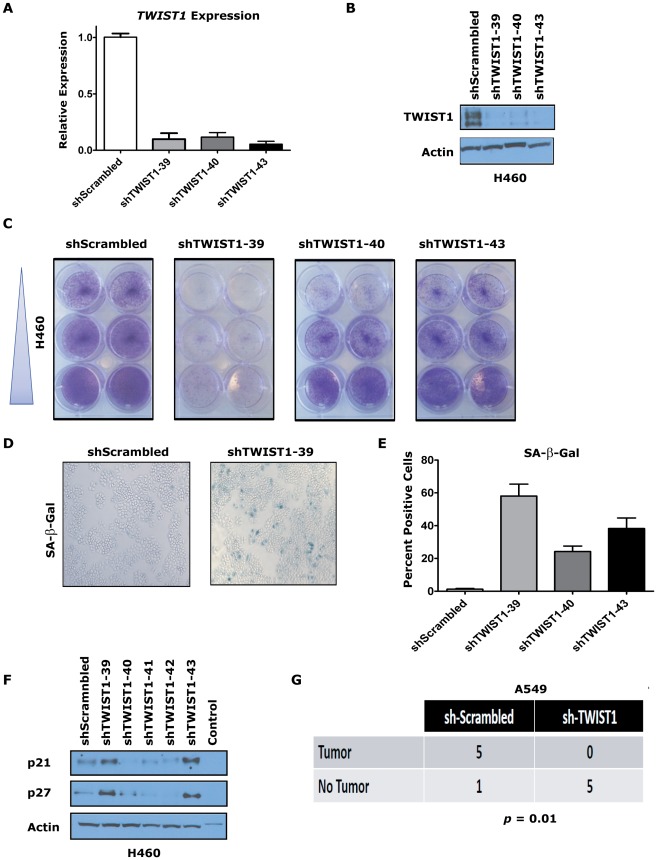
*TWIST1* knockdown activates senescence in human non-small cell lung cancer cells. Three different shRNAs were able to knockdown *TWIST1* mRNA levels and result in decreased TWIST1 protein in the *KRAS* mutated non-small cell lung cancer (NSCLC) cell line H460 as shown by (A) qPCR and (B) TWIST1 Western blotting on day 9 after the shRNA infection. (C) Representative duplicates of crystal violet staining of serially diluted H460 NSCLC cells demonstrate *TWIST1* knockdown decreases cellular proliferation. (D) Representative photomicrographs of increased SA-β-gal staining of cells following shRNA mediated *TWIST1* knockdown using sh-TWIST1-39. (E) Quantification of SA-β-gal stained cells following shRNA mediated *TWIST1* knockdown. (F) *TWIST1* knockdown in H460 results in the upregulation of some additional markers of senescence, p21 and p27 as shown by Western blotting on day 9 after the shRNA infection. (G) A549 cells require *TWIST1* overexpression to form subcutaneous tumors in NOD-SCID mice. A contingency table of A549 cells infected with sh-Scrambled control or sh-TWIST1 shRNA that were implanted into NOD-SCID mice and 4 weeks later scored for tumor development (5/6 *versus* 0/5, respectively, *p* = 0.01 by Fisher's exact test).

The overexpression of *TWIST1* in human lung cancers and our *in vivo* data from the CT-LSL OFF mouse lung tumors strongly suggested that *TWIST1* may be a relevant therapeutic target in human lung cancer. The consequences of knocking down *TWIST1* using shRNA technology was tested in human *KRAS* mutant H460 lung cancer cells. We screened various published shRNAs and found three sequences that were capable of knocking down human *TWIST1* as shown by qPCR (Figure 7A, *p*<0.029 by ANOVA) and at the protein level by Western (Figure 7B). *TWIST1* knockdown in H460 cells resulted in marked inhibition of proliferation using all three shRNAs (Figure 7C) and increased staining for the cellular senescence marker SA-β-Gal (Figure 7D–7E, *p*<0.023 by ANOVA). Other OIS markers p21 and p27 showed upregulation with a subset of the shRNAs examined (Figure 7F). We extended these results in two other human non-small cell lung cancer cell lines, H727 and A549, showing that *TWIST1* knockdown resulted in decreased proliferation and increased expression of markers consistent with activation of senescence (Figure S7).

We then confirmed that the *TWIST1* shRNA was not having off target effects by performing rescue experiments with mouse *Twist1* infected into H460 and A549 cells (Figure S8A and data not shown). Notably, the three shRNAs used in our study were not predicted to knockdown mouse *Twist1* cDNA, which was confirmed by qPCR (Figure S8B and data not shown). The anti-proliferative effects of shRNA mediated knockdown of human *TWIST1* in H460 and A549 cells was completely rescued by expression of mouse *Twist1* (Figure S8C and data not shown). These data provide evidence that inhibition of *TWIST1* can activate latent OIS in multiple different human *KRAS* mutant lung cancer cell lines.

To evaluate if the tumorigenic potential of human NSCLC cells required *TWIST1* overexpression, we performed subcutaneous xenografting experiments with A549 cells in immune-compromised NOD-SCID mice. A549 cell infected with sh-Scrambled control shRNA and subcutaneously injected into NOD-SCID mice produced large tumors with high efficiency (5/6 mice developed tumors by ≤4 weeks) necessitating humane euthanasia of the mice. In stark contrast, the identical experiment using sh-TWIST1 produced no tumors in any of the mice injected (Figure 7G, *p* = 0.015 by Fisher's exact test). These xenografting results confirm that *TWIST1* overexpression is required for tumorigenicity *in vitro* and *in vivo* in human NSCLC cells.

## Discussion

Our results dramatize that suppression of TWIST1 may be an effective pro-senescence therapy for human lung cancer. We provide the first *in vivo* demonstration that *Twist1* plays an important role in both the acceleration and maintenance of *Kras^G12D^*-induced autochthonous lung tumorigenesis. Our results illustrate that TWIST1 may be an important target for the treatment of human lung adenocarcinoma. We generated two novel autochthonous transgenic mouse models to demonstrate that *Twist1* overexpression cooperates with *Kras^G12D^* to markedly accelerate the onset of lung adenocarcinoma. Suppression of *Twist1* expression to physiological levels is sufficient to induce lung tumor stasis that was associated with the activation of cellular senescence programs. Importantly, through the transcriptional analysis of over 500 human tumors, human *TWIST1* was found to be frequently overexpressed and hence highly relevant to primary human lung cancers. Finally, the knockdown of *TWIST1* in human *KRAS* mutant lung tumor cells was also associated with the loss of their neoplastic properties and the induction of cellular senescence. The generality of our results using different cell types and across species suggest *TWIST1* is a potential therapeutic target in *KRAS* mutant lung cancers.

Oncogene-induced senescence and oncogene-induced apoptosis represent early tumor suppressive barriers that must be overcome for premalignant cells to ultimately emerge as neoplastic. It had been reported previously that *Twist1/2* could suppress mutant *Kras*-induced OIS *in vitro* [23], [24], but we report for the first time the ability of *Twist1* to suppress OIS *in vivo* using a novel *Twist1* lung model in combination with two complementary *Kras^G12D^*–induced autochthonous lung tumor models: the inducible transgenic *Kras^G12D^* (CR) model and the conditional endogenous *Kras^G12D^* (LSL) model. Our results are confirmed by an accompanying paper demonstrating that *Twist1* can also accelerate *Kras^G12D^*-induced autochthonous breast tumorigenesis (Morel et. al.). *Twist1* co-expression accelerated tumorigenesis relative to *Kras^G12D^* alone in both lung tumor models. *Twist1* acceleration was more pronounced in the CRT model than the CT-LSL model. One explanation for this difference is the greater strength of oncogenic signaling by transgenic *Kras^G12D^ versus* endogenous *Kras^G12D^* [53]. An alterative explanation is that cell type specific chromatin regulation of tumor suppressor loci such as the *Ink4a/Arf* locus is a key determinant of whether mutant *Kras* elicits tumor suppressive responses resulting in apoptosis and/or senescence [54]. Another explanation are strain difference effects as we had to use a mixed background for the CT-LSL mouse experiments. These alternatives are not mutually exclusive and further study using additional tissue specific models of *Kras^G12D^* and *Twist1* expression are needed to define the mechanistic basis for the differences we observed in oncogenic synergy observed between *Twist1* and *Kras^G12D^*.

The acceleration and progression of *Kras^G12D^* -induced lung tumors by *Twist1* is reminiscent of that seen with p53 deficiency [3], [44], [45], [55]. Notably, Twist1 may inhibit p53 through several independent mechanisms [23], [56], [57], [58], [59], [60], including direct Twist1-p53 antagonism [61]. One straightforward interpretation of our results is that *Twist1* overexpression can phenocopy *Trp53* deletion. *Twist1* may also accelerate and promote *Kras^G12D^*-induced lung tumors by the direct transcriptional regulation of *BMI1* [62]. As mentioned above, the control of tumor suppressor loci by chromatin regulatory complexes, such as those containing Bmi1, may be a strong determinant of responses to oncogenic signaling [54]. Interestingly, ectopic expression of *Twist1* in lung epithelial cells was associated with the induction of an EMT program. Whether the transdifferentiation program might contribute to accelerated tumor initiation, as proposed by Morel et. al., is also an intriguing possibility. Additional studies are required to define the mechanisms by which *Twist1* accelerates *Kras^G12D^*-induced lung tumors, as well as explain why different tissues exhibit differing cancer susceptibilities despite harboring the same initiating oncogenic event.

*Twist1* has been commonly implicated in metastasis [32]. Thus, our finding that *Twist1* expression did not seem to confer increase distant metastases in either the CRT or CT-LSL autochthonous lung tumor models was surprising. We note that *Twist1* appears to confer increased prometastatic ability in other models of tumorigenesis as predicted (D. I. Bellovin, P. T. Tran and D. W. Felsher, *unpublished data*). Hence, *Twist1* may have specific effects on metastatic potential.

Our study dramatically illustrates that it is possible to activate a latent senescence program in *Kras* mutant tumors *in vivo* by targeting the collaborating oncogene, *Twist1*. We uncover a newly defined synthetic interaction between mutant *Kras* and *Twist1* resulting not in cell death, but cellular senescence. The activation of this program is evident at the molecular level and most importantly results in marked inhibition of *Kras* mutant lung tumor growth *in vivo*. We realize that a possible caveat to this approach is that we first overexpressed *Twist1* prior to *Kras^G12D^* activation and lung tumor formation and thus may have biased tumors towards dependency for *Twist1*. However, simply overexpressing an oncogene during tumorigenesis does not *per se* make tumors dependent or “addicted” to that oncogene as we have shown, in particular for lung tumorigenesis [35], [63]. Finally, we validate that knocking down endogenous *TWIST1* in human lung cancer cell lines *in vitro* and *in vivo* also results in activation of senescence.

An alternative approach to inducible overexpression using the TET system as we used in our study would be to use genetic ablation of endogenous *Twist1* using the *Cre-LoxP* or a inducible shRNA system following development of *Kras^G12D^*–induced lung tumors. As *Kras^G12D^*–induced lung tumors are primarily adenomas with low proliferative rates (Figure 2E and Figure 4F), endogenous *Twist1* ablation or knockdown would not likely have an effect as has been shown for p53 restoration in adenomas [44], [45]. From a clinical standpoint complete ablation of a gene, such as in mice using the *Cre-LoxP* system, is therapeutically not possible in humans. In contrast, the TET model system where we can suppress *Twist1* overexpression to physiologic levels is more clinically relevant to what is done in the clinic with inhibitors. Others have shown senescence can arise *in vivo* in established tumors by targeting an initiating oncogene or reconstitution of a tumor suppressor [21], [64], [65], [66]. Our work further highlights the activation of a latent cellular senescence program or pro-senescence therapy as an innovative avenue for cancer therapy [67].

Our results may extend beyond *KRAS*-mutant lung cancers. Notably, *TWIST1* was found to be overexpressed in a majority of human lung cancer samples we tested. This includes not only adenocarcinoma, in which *KRAS* mutation is commonly observed, but also other major lung cancer histologies including squamous cell carcinoma, in which *KRAS* mutation is rare. Our preliminary data suggests that *TWIST1* knockdown can result in activation of OIS in *KRAS* wildtype lung cancer cell lines *in vitro*, but further characterization of these lines for mutations in other components of the EGFR/KRAS/BRAF pathway are needed (T.F. Burns, P. T. Tran and C. M. Rudin, *unpublished data*). Furthermore, additional preliminary findings suggest that *TWIST1* may have a larger role in suppressing OIS following activation of other key driver mutations using other transgenic mouse lines (P. T. Tran and D. W. Felsher, *unpublished data*). This hypothesis will be further explored in lung cancer through introduction of our inducible *Twist1* construct into other relevant transgenic models of lung tumorigenesis. Importantly, regardless of whether there is an exclusive association between *KRAS* mutation and *TWIST1* overexpression in human lung cancer cells, the data presented strongly support that TWIST1 upregulation in *KRAS* mutant lung cancer represents a novel and particularly promising therapeutic target. These observations have important and immediate translational implications for this particularly refractory subset of lung cancers.

The consequences of systemic transient inhibition of Twist1 in the adult has not been well defined and thus side-effects of such treatment are unknown. Germline deletion of *Twist1*1 in mice is embryonic lethal [22] and loss of function mutations in humans cause a severe developmental disorder. However, postnatal expression of *TWIST1* appears to be tightly restricted to a subpopulation of mesoderm derived tissues and limited studies suggest that Twist1 inhibition systemically may be well tolerated [68]. We conclude that *TWIST1* may be an effective target for “pro-senescence” therapy for human lung cancers [67]. Our results suggest that it will only be necessary to suppress TWIST1 to a physiological level which may preclude toxicity. Our mouse model will be useful to identify agents that target TWIST1 for the treatment of human cancer.

## Materials and Methods

### Cell lines

The human non-small cell lung cancer cell lines, H460, H727 and A549; and embryonic kidney cell line HEK 293 T were obtained from ATCC and grown in media as recommended.

MEFs were isolated from E13.5 embryos and propagated as described previously [18]. MEFs were grown for two population doublings and then frozen for future experiments. MEFs were grown in DMEM plus 10% fetal calf serum.

### Transgenic mice

The *Twist1* cDNA was PCR cloned into the bidirectional *tetO_7_* vector S2f-*IM*C*g* [33] at *EcoR*I and *Not*I sites, replacing the *eGFP* ORF. The resultant construct, *Twist1-tetO_7_-luc*, was sequence confirmed, digested with *Kpn*I and *Xmn*I to release the bidirectional transgene and then used for injection of FVB/N pronuclei by the Stanford Transgenic Facility. We ultimately obtained three founders from 25 pups after screening by tail genotyping using PCR as described below. These three founders were mated to CCSP-rtTA mice to screen for functional *Twist1-tetO_7_-luc* founders. One founder failed to pass the transgene germline and one founder did not report inducible *Twist1* or *luc* expression. The remaining founder was used for all the experiments in this study.

We use the *β-actin-rtTA*, *CCSP-rtTA*, *tetO-Kras4b^G12D^* and *LSL-K ras^G12D^* transgenic lines [3], [34], [69]. *Twist1* and/or *K-ras^G12D^* expression was activated in the CT, CR, and CRT lung lines by administering doxycycline (Sigma) to the drinking water weekly [2 mg/mL] starting at the age of 3–5 weeks. The conditional *LSL-K ras^G12D^* lines were activated by intranasal delivery of adenoviral CMV-Cre [43]. All procedures were performed in accordance with APLAC protocols and animals were housed in a pathogen-free environment.

### PCR genotyping

DNA was isolated from mouse tails using the Qiaprep DNeasy kit (Qiagen). The *CCSP-rtTA*, *tetO-K-ras^G12D^* and *LSL-K ras^G12D^* transgenic lines were screened as described previously. The *Twist1-tetO_7_-luc* line was detected with the following primers: mTwist1-Luc.S2 5′- CCTTATGCAGTTGCTCTCCAG -3′ and mTwist1-Luc.AS2 5′- GCTTGCCTATGTTCTTTTGGA -3′. DNA was amplified using PCR and PCR products were resolved on a 2% agarose gel.

### SYBR-green quantitative RT–PCR

Total RNA was isolated from tissue using the Qiaprep RNAeasy Kit (Qiagen) according to the manufacturer's directions. Samples were treated with RQ1 RNase-Free DNase (Promega). cDNA was generated from 1 µg of total RNA using the Superscript II kit (Invitrogen Technologies). Control reactions were run without RT enzyme. 50 ng of cDNA equivalents were amplified for the transcript described below in an ABI-prism 7700 for 40 cycles using SYBR green PCR Master mix (Perkin Elmer Applied Biosystems). PCR reactions were performed in duplicate-triplicate in a final volume of 20 µL. Following amplification, the data was processed with the analysis program Sequence Detection Systems v2.2.2 (Perkin Elmer Applied Biosystems). For each sample, the level of RNA for the genes of interest was standardized to a housekeeping gene (ubiquitin or 18S rRNA) within that sample; subsequently, the level of a transcript of interest was normalized to the expression of that transcript from the appropriate comparator sample. Primers for qPCR are listed in the Text S1.

Human normal lung and lung tumor qPCR tissue arrays and *TWIST1* qPCR oligos were purchased from OriGene. All relevant clinical information can be found (http://www.origene.com/qPCR/Tissue-qPCR-Arrays.aspx).

### Immunoblot analysis

Cells were lysed on ice for 60 min in radioimmunoprecipitation assay buffer supplemented with protease and phosphatase inhibitors (Sigma-Aldrich) and clarified by centrifugation. Protein concentrations were determined by Bradford proteinassay (Bio-Rad Laboratories). Equal protein concentrations of each sample were run on NuPAGE bis-Tris gels (Invitrogen) and transferred to membranes. After being blocked with 5% dried milk in TBS containing 0.2% Tween 20, the filters were incubated with primary antibodies. The following primary antibodies were used: goat anti-Actin (C-11, Santa Cruz), mouse anti-Twist1 (TWIST2C1a, Santa Cruz), mouse anti-p21 (Ab-1, Calbiochem)), mouse monoclonal anti-p27 (F-8, Santa Cruz) After washing and incubation with horseradish peroxidase (HRP)-conjugated anti-Goat or anti-mouse IgG (Amersham), the antigen-antibody complexes were visualized by chemiluminescence (ECL detection system; Perkin Elmer).

### Histology and immunohistochemistry

Tissues were fixed in 10% buffered formalin for 24 h and then transferred to 70% ethanol until embedded in paraffin. Tissue sections 5 µm thick were cut from paraffin embedded blocks, placed on glass slides and hematoxylin and eosin (H&E) or Masson's trichrome staining was performed using standard procedures. Antibodies used in our study: p21, p27, p16, vimentin (BD Pharmingen) and E-Cadherin (Cell Signaling). We performed IHC, measured K-i67 and CC3-staining as described previously [35]. For immunofluorescence (IF), Alexa488-conjugated anti-mouse and Alexa594-conjugated anti-rabbit (1∶300 dilution, Invitrogen) were used as secondary antibodies and incubated at room temperature for 30 minutes. DAPI was used as a nuclear stain and slides were mounted in aqueous mounting media (Vector Laboratories).

For EMT IF analysis double immunofluorescence was used. Vimetin-expressing cells were labelled with Alexa488 (green) and E-cadherin-expressing cells were labeled with Alexa 594 (red). To quantify cells undergoing EMT, cells that were red(low)green(high) were manually counted. A minimum of seven different fields of view per section from greater than four different animals were analyzed in total.

### Lentiviral and retroviral experiments

293T cells were seeded (2.5×106 cells) in T25 flasks. shRNA constructs were obtained from the Broad RNAi Consortium. pLKO.1-shRNA scramble vector was used. Lentivirus was made using a three-plasmid system and infected using the TRC Library Production and Performance Protocols. Twenty-four hours after infection, cells were treated with 1 mg/ml puromycin and passaged once 80% confluent.

Retroviral production used ecotropic and amphotropic Phoenix packaging lines. Early passage MEFs were transduced with pWZL-Hygro vectors expressing *Hras^G12V^* or with empty vector for two successive times over a 36-h period and then followed by selection with hygromycin (100 µg/ml) for 4 days. Retroviral infections on H460 cells used pWZL-Hygro vector and pWZL-Hygro/mTwist1 constructs, for two successive times over a 36-h period and then followed by selection with hygromycin (250 µg/ml) for 4 days.

### Colony formation and proliferation assays

On Day 6 after infection with the indicated shRNA lentiviruses, cells were plated in 12-well plates at a density of 5E3, 10E3 and 15E3 cells/well. On Day 12, the cells were stained with crystal violet (0.5% in 95% ethanol).

Similar low passage MEFs were used for all proliferation assays. Retroviral infections were performed as above, selection carried out for 4 days and stably selected cells were plated and then treated with or without 2 µg/ml doxyxycline for proliferation assays (Day 1). Sets of cells were removed for trypsinization and counting every 4 days. Values are normalized with Day1 readings.

### SA-β-gal staining

Cells were washed twice with phosphate-buffered saline (PBS) and then fixed with PBS containing 2% formaldehyde and 0.2% glutaraldehyde for 5 min. The cells were then incubated at 37°C for 20 hr with staining solution (40 mM citric acid sodium phosphate, pH 6.0, 1 mg/ml 5-bromo-4-chloro-3-isolyl-β-D-galactoside [X-gal, Fisher], 5 mM potassium ferricyanide, 5 mM potassium ferrocyanide, 150 mM NaCl, 2 mM MgCl_2_). After incubation, cells were washed twice with PBS and viewed with bright-field microscopy.

### Small animal imaging

Micro-computed tomography (μCT) and PET scans were performed on a custom GEHC (London, Ontario) eXplore RS150 cone-beam scanner and an R4 microPET (Siemens Medical Solutions USA, Inc.), respectively, as described previously [35], [70]. Mice were screened serially every 1–2 weeks following doxycycline activation or intranasal adenoviral CMV-Cre and images were reviewed by a board certified radiation oncologist (PTT). PET images were reconstructed using the ordered-subsets expectation maximization algorithm with a spatial resolution of 1.66 to 1.85 mm. No attenuation correction or partial volume corrections were applied.

### Lung tumor quantification

Micro-computed tomography (μCT) images were reviewed by a board certified radiation oncologist (PTT) on multiple index tumors in a blinded fashion (n = 2–5 tumors per mouse). Bi-dimensional measurements were made on tumors using serial examinations and tumor volumes calculated using the following equation vol = pi/6×1.65(length×width)×3/2. Volumes were normalized to the starting volume, t = 0 before doxycycline treatment, and percent tumor volume growth was then calculated by (normalized tumor vol.×100%)−100%.

### Mouse xenograft model

Female NOD-SCID mice 4–5 weeks old were purchased from Harlan Laboratories. Mice were maintained under pathogen-free conditions and given food and water *ad libitum* in accordance with guidelines from the Johns Hopkins Animal Care and Use Committee. A549 infected with sh-Scrambled control or sh-TWIST1 shRNA, selected for 4 days as described above and then 5×10^5^ million cells in 100 µL of Hank's solution and Matrigel (BD Biosciences) mixed 1∶1 were injected subcutaneously in the right flank. Tumor measurements were taken every 2–3 days.

## Supporting Information

Figure S1 Inducible *Twist1* lung model of epithelial mesenchymal transition (EMT). (A) Heatmap of the lung mRNA samples taken from CT mouse lungs Dox ON (n = 2) and wildtype mouse lungs Dox ON (n = 2) for the EMT_UP geneset. Enrichment plots for (B) HYPOXIA_NORMAL_UP, (C) HYPOPHARYNGEAL_MET_VS_NON_UP and (D) PMNS_DN following GSEA performed on CT ON lung samples and wildtype mouse lung samples (NOM *p*-values, FDR *q*-values, and FWER *p*-values were all <0.001 for all three genesets). (E) Representative immunofluorescence (IF) for the EMT markers E-cadherin and vimentin on lungs of CT and wildtype mice that was used for quantification of Figure 1E.
(TIF)

Figure S2 *Twist1* accelerates *Kras^G12D^*-induced lung tumorigenesis and promotes progression to adenocarcinoma. (A) Representative Ki-67 staining of lung tumors from CR and CRT mice used for quantification of Figure 2E.
(TIF)

Figure S3 *Kras^G12D^*/*Twist1*-induced lung tumors regress following combined oncogene inactivation. (A) Serial FDG microPET-CT volumetric reconstructions demonstrate decreased metabolic tumor burden after only 1 week of combined *Kras^G12D^* and *Twist1* oncogene inactivation (representative of n = 2). (B) Representative H&E and Masson's trichrome staining of CRT OFF lungs show fibrotic scars are present at the sites of presumed lung tumor regression. Black bars equal 50 µm. (C) Representative Ki-67 IF staining of lung tumors from CRT mice following combined *Kras^G12D^* and *Twist1* oncogene inactivation for the indicated time. (D) Representative cleaved caspase 3 (CC3) IHC staining of lung tumors from CRT mice following combined *Kras^G12D^* and *Twist1* oncogene inactivation for the indicated time. Black arrows denote CC3 positive staining cells.
(TIF)

Figure S4 Activation of *ras*-induced senescence by inactivation of *Twist1* in mouse embryonic fibroblasts (MEFs). (A) Western blot of *β-actin-rtTA/Twist1-tetO_7_-luc* MEFs used in the study demonstrating inducible Twist1 expression *in vitro*. Blots were probed with a Twist1 immunoreactive antibody and then stripped and reprobed with actin to ensure equal loading. (B) Representative growth curves of inducible *β-actin-rtTA/Twist1-tetO_7_-luc* MEFs infected with virus containing a control vector *versus Hras^G12V^* and then induced with doxycycline (+Dox) or without doxycycline (−Dox). Growth was normalized to Day 1. (C) Representative photomicrographs of senescence associated-β-galactosidase (SA-β-gal) staining of the cells in (B) at day 12. (D) Quantification of the SA-β-gal-positive percentage of cells in (C), *p* = 0.0286 by *t*-test (for both *Twist1*+Vector+Dox *versus Twist1*+*Hras^G12V^*−Dox and *Twist1*+*Hras^G12V^*+Dox *versus Twist1*+*Hras^G12V^*−Dox). (E) Deinduction of *Twist1* activates senescence as shown by cells from (B) at Day 12 that had doxycycline removed or continued in the media and then cell number counted 8 days later, *p* = 0.0025 by paired *t*-test. (F) SA-β-gal staining and quantification of the SA-β-gal-positive percentage of cells in (E), *p* = 0.0294 by *t*-test.
(TIF)

Figure S5 *Twist1* inactivation in the setting of *Kras* mutation results in gene expression changes consistent with an ectopic p21 overexpression gene expression signature. The mRNA was purified from CT-LSL ON (n = 2) and CT-LSL OFF (n = 5) mice and then subjected to microarray gene expression analysis. (A) Heatmap of the top 25 up- and down-regulated genes between CT-LSL ON *versus* CT-LSL OFF (*t*-test>5). (B) Additional mRNA was purified from normal lung (n = 2) and microdissected tumors from CR (n = 2), CRT (n = 2) and LSL (n = 2) mice and then subjected to microarray gene expression analysis. Single sample GSEA (ssGSEA) was used in preference over traditional GSEA as this new technique allows more robust analysis from limited sample sets (Barbie et al. 2009). The ssGSEA heat map of the top 25 correlated gene sets for normal lung, CR, CRT, LSL, CT-LSL ON and CT-LSL OFF samples reveals enrichment of p21_ANY and p21_ANY_UP gene set (boxed) in CT-LSL OFF relative to CT-LSL ON tumors. A figure incorporating all these samples (normal lung, CR, CRT, LSL, CT-LSL ON and CT-LSL OFF) was too cumbersome to present in its entirety, for the sake of clarity we only present the CT-LSL ON *versus* CT-LSL OFF portion of the results.
(TIF)

Figure S6 *Twist1* inactivation in the setting of *Kras* mutation results in gene expression changes that effect multiple canonical pathways specifically those for cell cycle arrest and senescence. The mRNA was purified from CT-LSL ON (n = 2) and CT-LSL OFF (n = 5) mice and then subjected to microarray gene expression analysis. Ingenuity Pathway Analysis software v5.0 (IPA) was utilized to identify the top significant canonical pathways from differentially expressed genes and their fold changes. The most significant network of probe sets constructed using IPA 5.0 is represented as nodes and lines between two nodes. Node shapes: square, cytokine; diamond, enzyme; inverted triangle, kinase; rectangle, nuclear receptor; ellipse, transcription regulator; circle, other. The intensity of node colors indicates the degree of upregulation (red) or downregulation (green). Continuous and dashed lines indicate direct and indirect interactions between molecules, respectively. Selected interesting genes are highlighted by blue ovals. (A) IPA analysis of CT-LSL ON. (B) CT-LSL OFF.
(TIF)

Figure S7 *TWIST1* knockdown activates senescence *in vitro* in H727 and A549 human non-small cell lung cancer lines. (A) The shRNAs shTWIST1-39 and -43 were able to knockdown *TWIST1* mRNA levels as shown by qPCR at day 4 after the shRNA infection. (B) Representative triplicates of crystal violet staining of H727 and A549 NSCLC cells demonstrate *TWIST1* knockdown decreases cellular proliferation. (C) *TWIST1* knockdown in H727 and A549 results in the upregulation of markers of senescence, p21, p27 and dephosphorylated pRb as shown by Western blotting on day 9 after the shRNA infection.
(TIF)

Figure S8 Mouse *Twist1* can rescue the anti-proliferative effects of knockdown of human *TWIST1* in H460 cells. (A) Twist1 Western blot of H460 cells stably infected with mouse *Twist1*. (B) Knockdown of human *TWIST1* mRNA but not mouse *Twist1* mRNA using human specific shRNAs in stably infected H460 cells from (A) as shown by qPCR. (C) Mouse *Twist1* rescues the anti-proliferative phenotype of human *TWIST1* knockdown in H460 cells as shown by crystal violet staining of cells in triplicate.
(TIF)

Text S1 Supporting information texts. Microarray Analysis, Gene Set Enrichment Analysis and Ingenuity Pathway Analysis methods and oligo sequences for qPCR are provided.
(DOC)
