# Supplementary figures and images for: Twist1 Suppresses Senescence Programs and Thereby Accelerates and Maintains Mutant Kras-Induced Lung Tumorigenesis
Source: PLoS Genet. 2012 May 24;8(5):e1002650. doi: 10.1371/journal.pgen.1002650 (PMC3360067; doi:10.1371/journal.pgen.1002650)

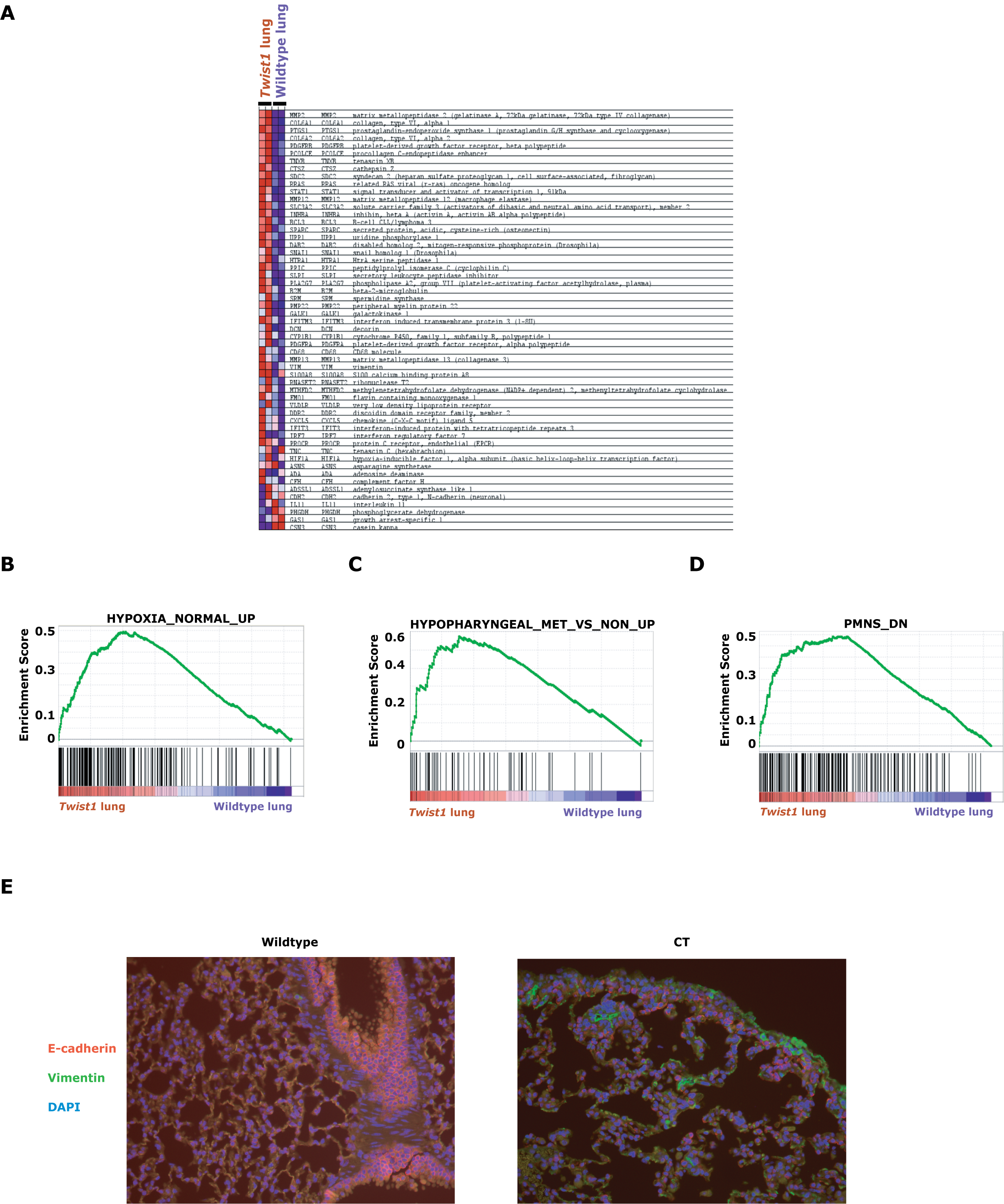

Supplement: Figure S1 — Inducible Twist1 lung model of epithelial mesenchymal transition (EMT). (A) Heatmap of the lung mRNA samples taken from CT mouse lungs Dox ON (n = 2) and wildtype mouse lungs Dox ON (n = 2) for the EMT_UP geneset. Enrichment plots for (B) HYPOXIA_NORMAL_UP, (C) HYPOPHARYNGEAL_MET_VS_NON_UP and (D) PMNS_DN following GSEA performed on CT ON lung samples and wildtype mouse lung samples (NOM p-values, FDR q-values, and FWER p-values were all <0.001 for all three genesets). (E) Representative immunofluorescence (IF) for the EMT markers E-cadherin and vimentin on lungs of CT and wildtype mice that was used for quantification of Figure 1E. (TIF) [file pgen.1002650.s001.tif]

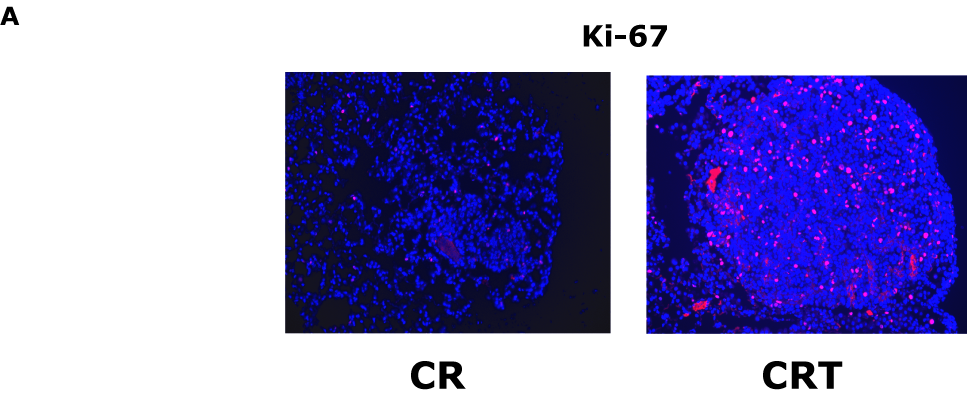

Supplement: Figure S2 — Twist1 accelerates KrasG12D-induced lung tumorigenesis and promotes progression to adenocarcinoma. (A) Representative Ki-67 staining of lung tumors from CR and CRT mice used for quantification of Figure 2E. (TIF) [file pgen.1002650.s002.tif]

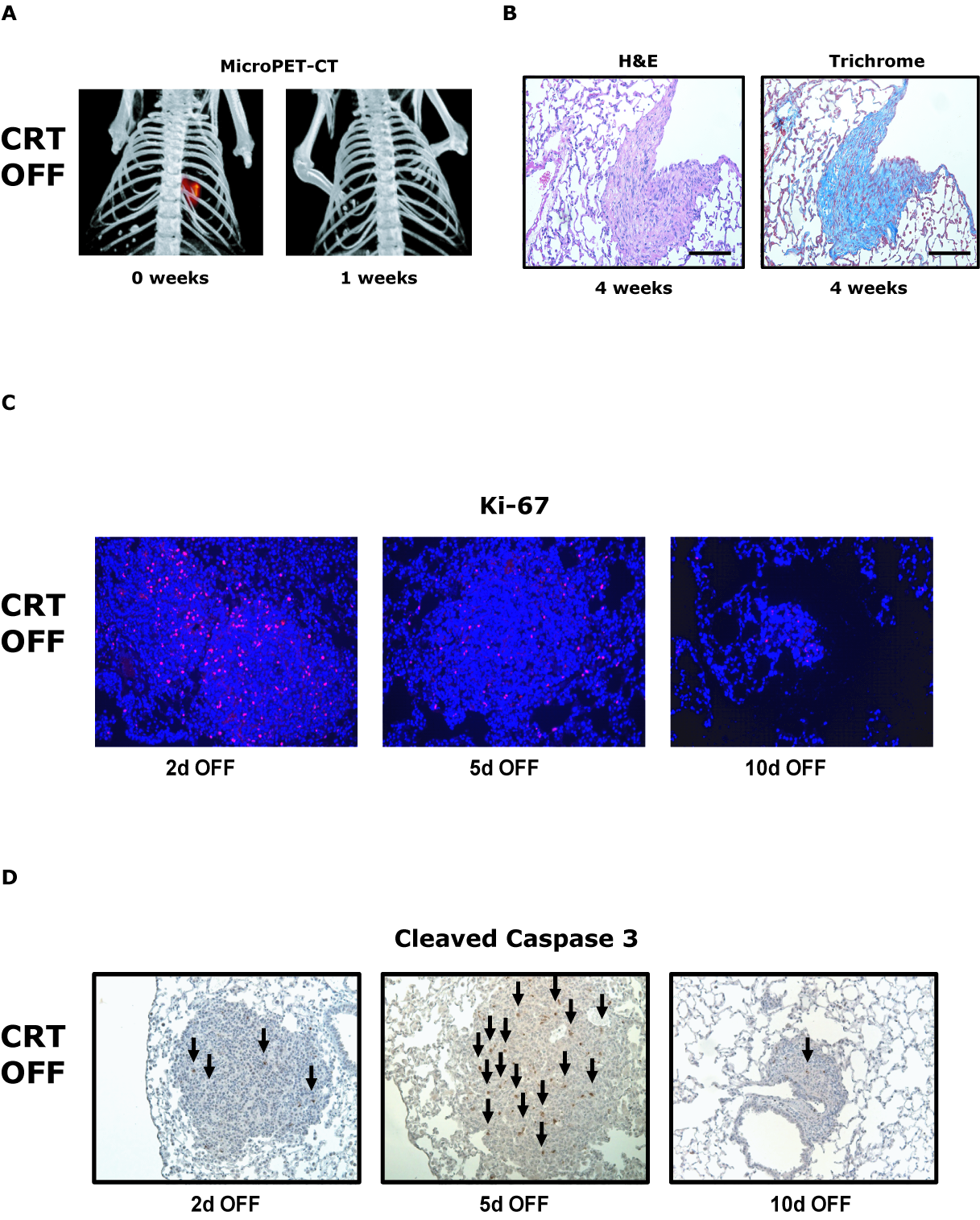

Supplement: Figure S3 — KrasG12D/Twist1-induced lung tumors regress following combined oncogene inactivation. (A) Serial FDG microPET-CT volumetric reconstructions demonstrate decreased metabolic tumor burden after only 1 week of combined KrasG12D and Twist1 oncogene inactivation (representative of n = 2). (B) Representative H&E and Masson's trichrome staining of CRT OFF lungs show fibrotic scars are present at the sites of presumed lung tumor regression. Black bars equal 50 µm. (C) Representative Ki-67 IF staining of lung tumors from CRT mice following combined KrasG12D and Twist1 oncogene inactivation for the indicated time. (D) Representative cleaved caspase 3 (CC3) IHC staining of lung tumors from CRT mice following combined KrasG12D and Twist1 oncogene inactivation for the indicated time. Black arrows denote CC3 positive staining cells. (TIF) [file pgen.1002650.s003.tif]

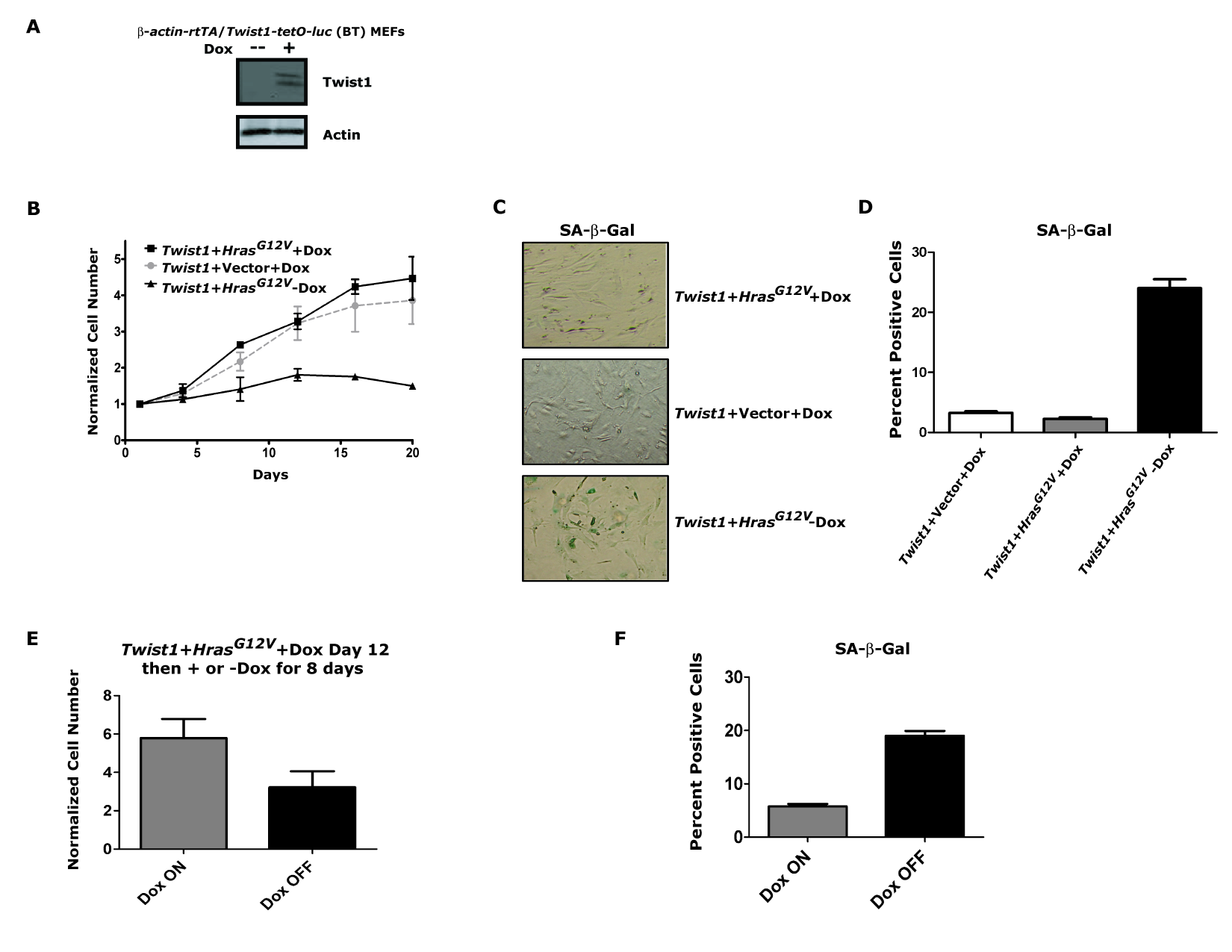

Supplement: Figure S4 — Activation of ras-induced senescence by inactivation of Twist1 in mouse embryonic fibroblasts (MEFs). (A) Western blot of β-actin-rtTA/Twist1-tetO7-luc MEFs used in the study demonstrating inducible Twist1 expression in vitro. Blots were probed with a Twist1 immunoreactive antibody and then stripped and reprobed with actin to ensure equal loading. (B) Representative growth curves of inducible β-actin-rtTA/Twist1-tetO7-luc MEFs infected with virus containing a control vector versus HrasG12V and then induced with doxycycline (+Dox) or without doxycycline (−Dox). Growth was normalized to Day 1. (C) Representative photomicrographs of senescence associated-β-galactosidase (SA-β-gal) staining of the cells in (B) at day 12. (D) Quantification of the SA-β-gal-positive percentage of cells in (C), p = 0.0286 by t-test (for both Twist1+Vector+Dox versus Twist1+HrasG12V−Dox and Twist1+HrasG12V+Dox versus Twist1+HrasG12V−Dox). (E) Deinduction of Twist1 activates senescence as shown by cells from (B) at Day 12 that had doxycycline removed or continued in the media and then cell number counted 8 days later, p = 0.0025 by paired t-test. (F) SA-β-gal staining and quantification of the SA-β-gal-positive percentage of cells in (E), p = 0.0294 by t-test. (TIF) [file pgen.1002650.s004.tif]

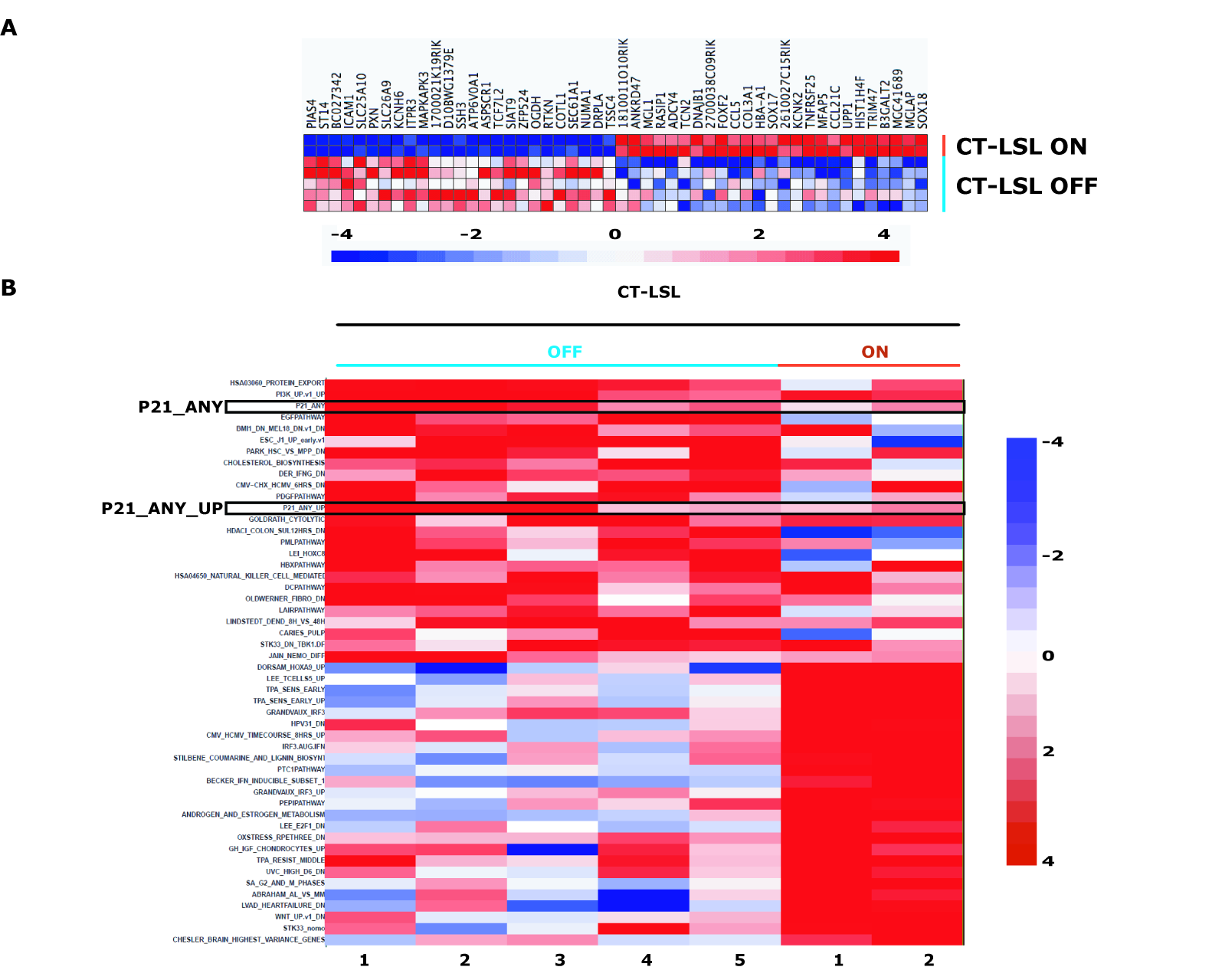

Supplement: Figure S5 — Twist1 inactivation in the setting of Kras mutation results in gene expression changes consistent with an ectopic p21 overexpression gene expression signature. The mRNA was purified from CT-LSL ON (n = 2) and CT-LSL OFF (n = 5) mice and then subjected to microarray gene expression analysis. (A) Heatmap of the top 25 up- and down-regulated genes between CT-LSL ON versus CT-LSL OFF (t-test>5). (B) Additional mRNA was purified from normal lung (n = 2) and microdissected tumors from CR (n = 2), CRT (n = 2) and LSL (n = 2) mice and then subjected to microarray gene expression analysis. Single sample GSEA (ssGSEA) was used in preference over traditional GSEA as this new technique allows more robust analysis from limited sample sets (Barbie et al. 2009). The ssGSEA heat map of the top 25 correlated gene sets for normal lung, CR, CRT, LSL, CT-LSL ON and CT-LSL OFF samples reveals enrichment of p21_ANY and p21_ANY_UP gene set (boxed) in CT-LSL OFF relative to CT-LSL ON tumors. A figure incorporating all these samples (normal lung, CR, CRT, LSL, CT-LSL ON and CT-LSL OFF) was too cumbersome to present in its entirety, for the sake of clarity we only present the CT-LSL ON versus CT-LSL OFF portion of the results. (TIF) [file pgen.1002650.s005.tif]

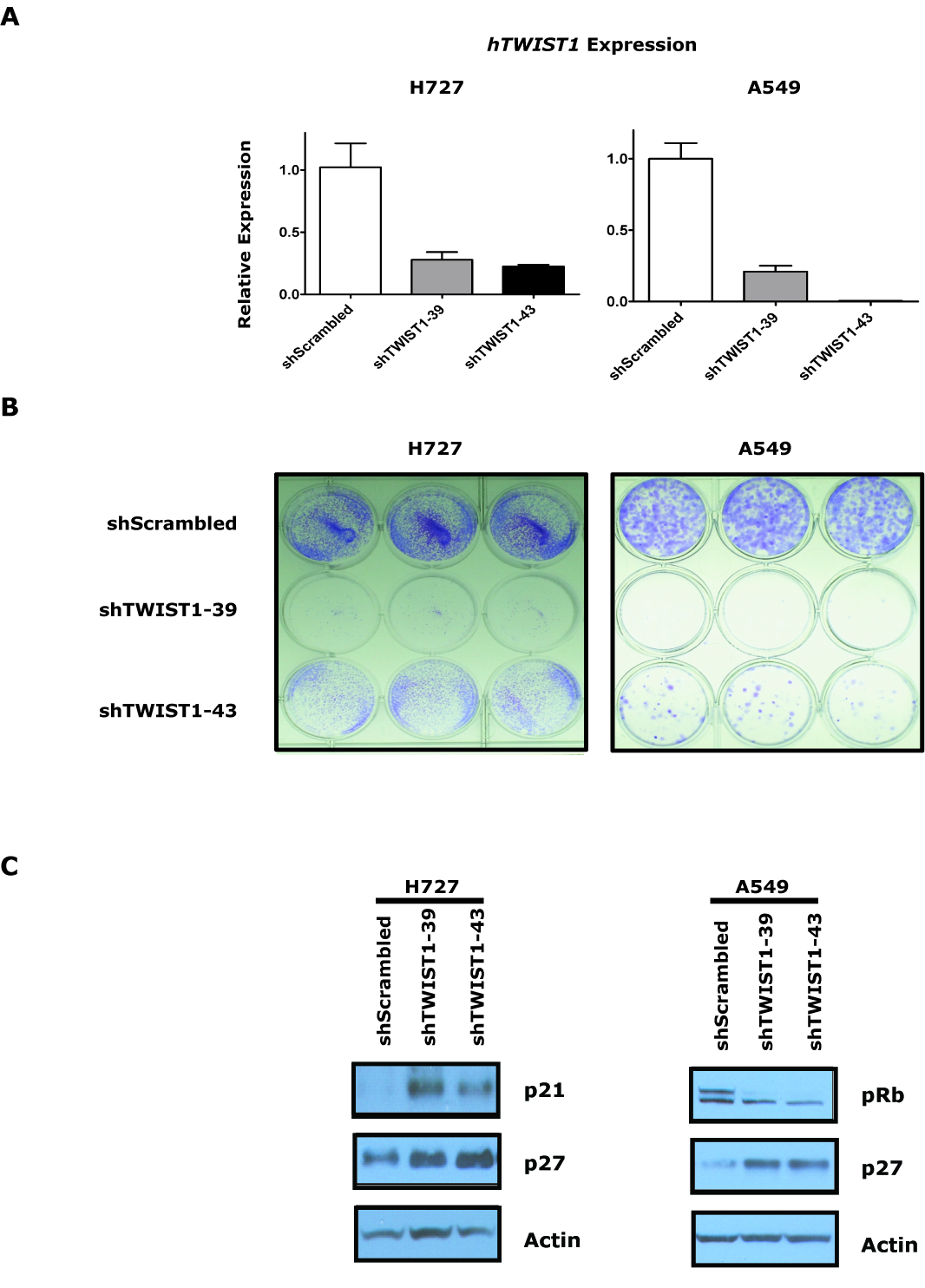

Supplement: Figure S7 — TWIST1 knockdown activates senescence in vitro in H727 and A549 human non-small cell lung cancer lines. (A) The shRNAs shTWIST1-39 and -43 were able to knockdown TWIST1 mRNA levels as shown by qPCR at day 4 after the shRNA infection. (B) Representative triplicates of crystal violet staining of H727 and A549 NSCLC cells demonstrate TWIST1 knockdown decreases cellular proliferation. (C) TWIST1 knockdown in H727 and A549 results in the upregulation of markers of senescence, p21, p27 and dephosphorylated pRb as shown by Western blotting on day 9 after the shRNA infection. (TIF) [file pgen.1002650.s007.tif]

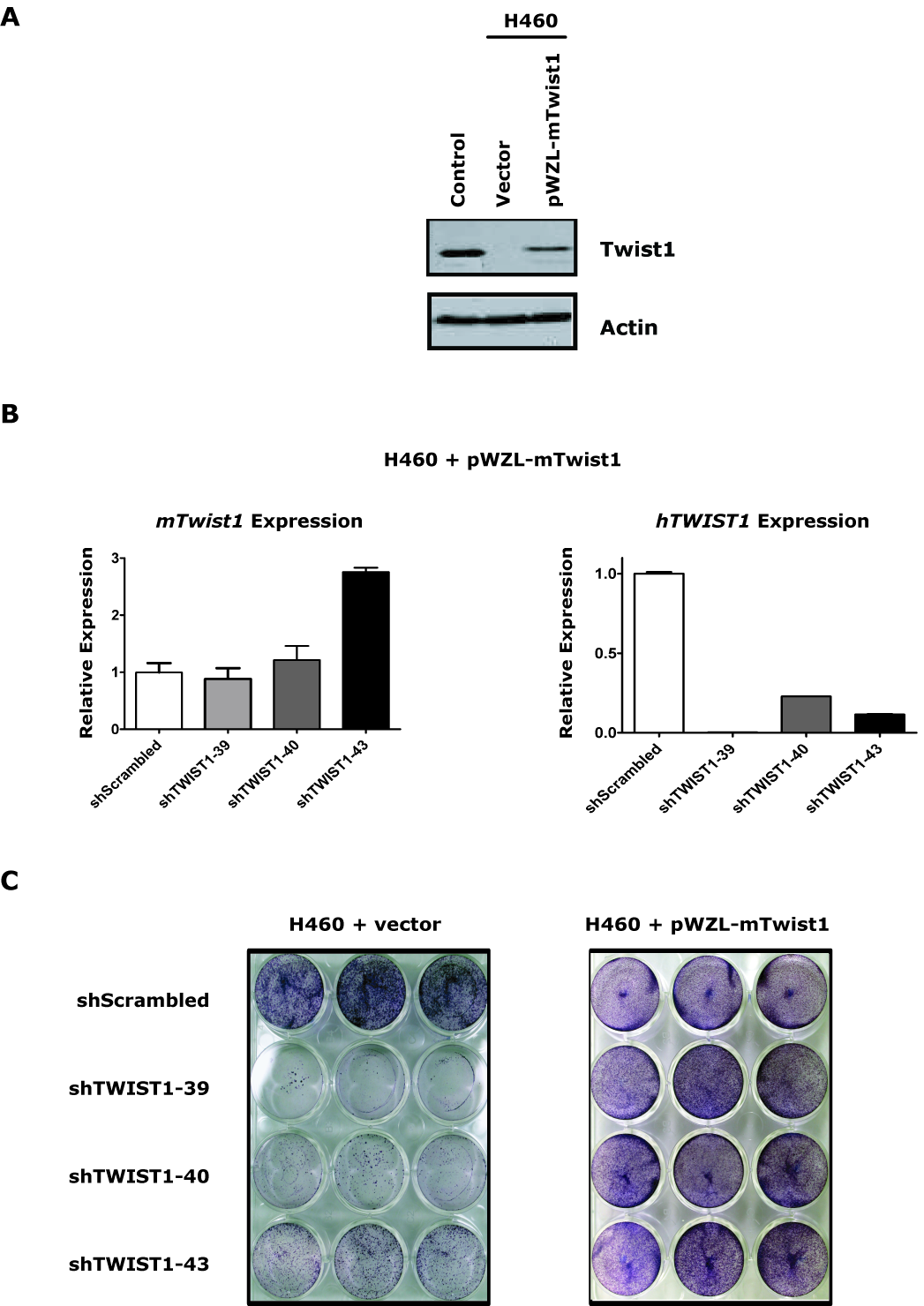

Supplement: Figure S8 — Mouse Twist1 can rescue the anti-proliferative effects of knockdown of human TWIST1 in H460 cells. (A) Twist1 Western blot of H460 cells stably infected with mouse Twist1. (B) Knockdown of human TWIST1 mRNA but not mouse Twist1 mRNA using human specific shRNAs in stably infected H460 cells from (A) as shown by qPCR. (C) Mouse Twist1 rescues the anti-proliferative phenotype of human TWIST1 knockdown in H460 cells as shown by crystal violet staining of cells in triplicate. (TIF) [file pgen.1002650.s008.tif]
