# Supplementary material for: Twist1 Suppresses Senescence Programs and Thereby Accelerates and Maintains Mutant Kras-Induced Lung Tumorigenesis
Source: PLoS Genet. 2012 May 24;8(5):e1002650. doi: 10.1371/journal.pgen.1002650 (PMC3360067; doi:10.1371/journal.pgen.1002650)
Supplement: Text S1 — Supporting information texts. Microarray Analysis, Gene Set Enrichment Analysis and Ingenuity Pathway Analysis methods and oligo sequences for qPCR are provided. (DOC) [file pgen.1002650.s009.doc]

**Text s1**

**Microarray Analysis, Gene Set Enrichment Analysis and Ingenuity Pathway Analysis**

Snap frozen samples of CT lungs, wildtype lungs and CR, CRT and CT-LSL microdissected lung tumors as stated in the text were obtained at necropsy. Total RNA was isolated as described for qPCR, applied to Illumina arrays, files preprocessed and normalized using default parameters in the Genespring GX 11 software package (Agilent, Santa Clara, CA) and/or the DASL pipeline in Genepattern (www.broadinstitute.org/genepattern). Arrays were visualized using Genespring GX 11 or GenePattern software packages. Gene set enrichment analysis (GSEA) was performed on wildtype lungs and CT lungs as previously described {Subramanian et. al. 2005} using a reference database of 2,163 gene sets: 1,891 gene sets from the MSigDB-C2 database [http://www.broad.mit.edu/gsea/msigdb] and 272 additional manually curated gene sets representing oncogene activation/tumor suppressor deregulation (OPAM database v3) {Barbie et. al. 2009}. To all gene sets we added sets that combined up and down regulated sets derived from the same experimental condition or publication. The final total was 2,599 signatures.

The ‘single sample’ gene set enrichment analysis (ssGSEA) was performed on normal lung (n=2) and microdissected tumors from CR (n=2), CRT (n=2), LSL (n=2), CT-LSL ON (n=2) and CT-LSL OFF (n=5) mice. We defined a numerical score to represent the single-sample absolute enrichment in each of the samples for each of gene set of interest using the following procedure. Probes in each pre-processed gene-expression dataset were mapped to gene symbols using the probe “collapse” feature from GSEA analysis [www.broad.mit.edu/gsea] and the genes ranked by absolute expression for each sample. The enrichment score is produced by evaluating a weighted integral (sum) of the difference between the Empirical Cumulative Distribution Functions (ECDF) of the genes in the gene set vs. genes not in the set, rather than the maximum (Kolmogorov- Smirnov ) as used in GSEA. For gene sets with up-regulated (UP) and down-regulated (DN) versions, a combined score is produced by adding the UP and DN scores (with negative sign). To quantify the degree of matching between phenotypes vs. pathway profiles we used the area under the ROC (Receiver Operating Characteristic ) curve ranging from 0.5 (random match) to 1.0 (perfect match). The statistical significance is computed using a binomial test. Both computations use the R “verification” package.

 Ingenuity Pathway Analysis software (IPA 5.0; Ingenuity Systems, Redwood City, CA) was also utilized to identify the top significant canonical pathways for microdissected tumors from CT-LSL ON (n=2) and CT-LSL OFF (n=5) mice. IPA was used to study the gene signaling pathways that were involved in biological processes when Twist1 was inactivated in our CT-LSL tumors. Differentially expressed genes and their fold changes were uploaded into IPA for analysis.

**Oligos for qPCR**

Transgenic *K-ras* exon 4b

K-ras4b-fwd 5’- CAAGGACAAGGTGTACAGTTATGTGACT-3’

mp-1-real time-rev 5’-GGCATCTGCTCCTGCTTTTG-3’

Endogenous *K-ras4b* 3UTR

K-ras-4b-UTR-fwd 5’-GCAGGGTTGGGCCTTACAT-3’

K-ras-4b-UTR rev 5’-ATGCGTCGCCACATTGAAT-3’

18S rRNA

forward 5’-AACGAACGAGACTCTGGCAT-3’

reverse 5’-CAAGCTTATGACCCGCACTT-3’

*Dec1*

forward 5’-GGCGGGGAATAAAACGGAGCGA-3’

reverse 5’-CCTCACGGGCACAAGTCTGGAA-3’

*mCdkn1a* (p21)

forward 5’-CCAGGCCAAGATGGTGTCTT-3’

reverse 5’-TGAGAAAGGATCAGCCATTGC-3’

*hCDKN1A* (p21)

forward 5’-GAGAGCGCAGACTACTGCATC-3’

reverse 5’-CCACGAAAAGGGCATTAGGAAAA-3’

*mCdkn2a* (p16)

forward 5’-CGACGGGCATAGCTTCAG-3’

reverse 5’-ACGCTAGCATCGCTAGAAGTG-3’

*hCDKN2A*(p16)

forward 5’-GGGTTTTCGTGGTTCACATCC-3’

reverse 5’-CTAGACGCTGGCTCCTCAGTA-3’

*TP53* (p53)

forward 5’-CCAGGGCAGCTACGGTTTC-3’

reverse 5’-CTCCGTCATGTGCTGTGACTG-3’

*p19ARF*

forward 5’-CACTGTGAGGATTCAGCG-3’

reverse 5’-CATCATCATCACCTGGTCC-3’

*ubiquitin*

ubiquitin forward 5’-AGCCCAGTGTTACCACCAAG-3’

ubiquitin reverse 5’-ACCCAAGAACAAGCACAAGG-3’

*mTwist1*

forward 5’-GGACAAGCTGAGCAAGATTCA-3’

reverse 5’-CGGAGAAGGCGTAGCTGAG-3’

*hTWIST1*

forward 5’-GCTTGAGGGTCTGAATCTTGCT-3’

reverse 5’-GTCCGCAGTCTTACGAGGAG-3’

**text S1 References**

Barbie *et. al.,* *Nature.* 462, 108 (2009).

Subramanian *et. al.,* *Proc Natl Acad Sci U S A.* 102, 15545 (2005).
